# Molecular profiling reveals features of clinical immunity and immunosuppression in asymptomatic *P. falciparum* malaria

**DOI:** 10.15252/msb.202110824

**Published:** 2022-04-27

**Authors:** Stephanie I Studniberg, Lisa J Ioannidis, Retno A S Utami, Leily Trianty, Yang Liao, Waruni Abeysekera, Connie S N Li‐Wai‐Suen, Halina M Pietrzak, Julie Healer, Agatha M Puspitasari, Dwi Apriyanti, Farah Coutrier, Jeanne R Poespoprodjo, Enny Kenangalem, Benediktus Andries, Pak Prayoga, Novita Sariyanti, Gordon K Smyth, Alan F Cowman, Ric N Price, Rintis Noviyanti, Wei Shi, Alexandra L Garnham, Diana S Hansen

**Affiliations:** ^1^ The Walter and Eliza Hall Institute of Medical Research Parkville Vic. Australia; ^2^ Department of Medical Biology The University of Melbourne Parkville Vic. Australia; ^3^ Eijkman Institute for Molecular Biology Jakarta Indonesia; ^4^ Olivia Newton‐John Cancer Research Institute Heidelberg Vic. Australia; ^5^ School of Mathematics and Statistics The University of Melbourne Parkville Vic. Australia; ^6^ Papuan Health and Community Foundation Papua Indonesia; ^7^ Global and Tropical Health Division Menzies School of Health Research and Charles Darwin University Darwin NT Australia; ^8^ Centre for Tropical Medicine and Global Health Nuffield Department of Medicine University of Oxford Oxford UK; ^9^ Mahidol‐Oxford Tropical Medicine Research Unit Mahidol University Bangkok Thailand

**Keywords:** asymptomatic infection, immunity, immunosuppression, malaria, *P. falciparum*, Immunology, Microbiology, Virology & Host Pathogen Interaction, Molecular Biology of Disease

## Abstract

Clinical immunity to *P. falciparum* malaria is non‐sterilizing, with adults often experiencing asymptomatic infection. Historically, asymptomatic malaria has been viewed as beneficial and required to help maintain clinical immunity. Emerging views suggest that these infections are detrimental and constitute a parasite reservoir that perpetuates transmission. To define the impact of asymptomatic malaria, we pursued a systems approach integrating antibody responses, mass cytometry, and transcriptional profiling of individuals experiencing symptomatic and asymptomatic *P. falciparum* infection. Defined populations of classical and atypical memory B cells and a T_H2_ cell bias were associated with reduced risk of clinical malaria. Despite these protective responses, asymptomatic malaria featured an immunosuppressive transcriptional signature with upregulation of pathways involved in the inhibition of T‐cell function, and CTLA‐4 as a predicted regulator in these processes. As proof of concept, we demonstrated a role for CTLA‐4 in the development of asymptomatic parasitemia in infection models. The results suggest that asymptomatic malaria is not innocuous and might not support the induction of immune processes to fully control parasitemia or efficiently respond to malaria vaccines.

## Graphical abstract

Asymptomatic *Plasmodium falciparum* malaria infection supports protective humoral responses, but it also features an immunosuppressive transcriptional signature with upregulation of pathways involved in the inhibition of T‐cell function.

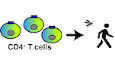

## Introduction

Malaria remains one of the most serious infectious diseases of humans with over 200 million clinical cases and 600,000 deaths estimated in 2020 (World Health Organization, 2021). *Plasmodium falciparum* is the most virulent species of malaria parasites and is responsible for disease syndromes ranging from febrile illness to life‐threatening complications including respiratory distress, hypoglycemia, renal failure, pulmonary edema, and cerebral malaria (White & Ho, 1992; Miller *et al*, 2002). A large body of data supports the concept that disease syndromes result from the sequestration of parasitized red blood cells (RBCs) in vascular beds of target organs (Miller *et al*, 2002). Inflammatory responses also contribute to severe disease, with high levels of TNF (Molyneux *et al*, 1993), IFN‐γ, IL‐1β (Pongponratn *et al*, 2003), and CXCL10 (Wilson *et al*, 2011) found to be associated with severe *P. falciparum* malaria. Immune mechanisms controlling parasite growth to below the threshold inducing clinical symptoms develop after repeated exposure over several years. Antibodies are important in clinical immunity to malaria with roles that include inhibition of parasite invasion into RBCs (Blackman *et al*, 1990) and opsonization for phagocytosis (Hill *et al*, 2013).

The acquisition of long‐lived antibody‐mediated immunity requires the establishment of germinal centers (GCs) in secondary lymphoid organs, where naive B cells activated by cognate antigen undergo somatic hypermutation of their immunoglobulin (Ig) genes followed by selection of B‐cell clones of high affinity for antigen. These processes require help from T follicular helper (T_FH_) cells (Vinuesa *et al*, 2005), which orchestrate GC responses and promote the differentiation of B cells into long‐lived plasma cells and memory B cells (MBCs) (Crotty, 2014). Recent studies in infection models and human malaria revealed that inflammatory cytokines produced in response to acute malaria such as IFN‐γ play an important role in modulating the development of these processes, by upregulating expression of the T helper 1 (T_H1_)‐defining transcription factor T‐bet in T_FH_ cells and GC B cells. While T‐bet expression in T_FH_ cells impairs their differentiation (Ryg‐Cornejo *et al*, 2016), thereby reducing the magnitude of the antibody response (Obeng‐Adjei *et al*, 2015; Ryg‐Cornejo *et al*, 2016), T‐bet expression in B cells promotes the differentiation of cells with increased affinity for antigen, thereby improving the quality of the antibody response (Ly *et al*, 2019). Thus, the same inflammatory pathways mediating disease symptoms modulate the acquisition of antibody‐mediated clinical immunity.

Clinical immunity to malaria is not sterilizing, and adults in endemic areas often experience asymptomatic infections. Field studies have shown that antibodies to *P. falciparum* antigens are rapidly lost in the absence of ongoing exposure to the parasite (Fruh *et al*, 1991; Kinyanjui *et al*, 2007; Weiss *et al*, 2010), suggesting that asymptomatic infections might be required to sustain clinical immunity (Kinyanjui *et al*, 2004). Aligned with that view, the presence of asymptomatic *P. falciparum* infections at the end of the dry season was found to reduce the risk of febrile illness in the ensuing malaria season (al‐Yaman *et al*, 1997; Bereczky *et al*, 2007; Doumbo *et al*, 2014). Thus, despite perpetuating a parasite reservoir that deters malaria elimination efforts (Schneider *et al*, 2007; Sattabongkot *et al*, 2018), asymptomatic malaria has been long viewed as beneficial to help reduce the risk of severe disease. However, emerging evidence suggests that persistent asymptomatic malaria infections could be detrimental, with important health, developmental, and productivity consequences (Chen *et al*, 2016). Furthermore, recent studies revealed that treatment of asymptomatic individuals before the start of transmission season does not increase their risk of symptomatic malaria upon re‐infection (Portugal *et al*, 2017), suggesting that subclinical infections might not always be required to maintain clinical immunity.

To date, the real impact that asymptomatic malaria has on the host and whether these infections should be treated or not remain controversial. To address these issues, we pursued a systems biology approach integrating antibody profiling, high‐dimensional mass cytometry, and peripheral blood mononuclear cell (PBMC) transcriptomic analysis in individuals from a malaria‐endemic area experiencing symptomatic and asymptomatic *P. falciparum* infection. Antibody responses to parasite invasion ligands, populations of classical and atypical MBCs as well as T helper 2 (T_H2_) cells were associated with a reduced risk of clinical malaria in asymptomatic individuals. Despite these protective responses, asymptomatic *P. falciparum* malaria was also characterized by a strong immunosuppressive transcriptional signature with the upregulation of several inhibitory pathways and featuring cytotoxic T‐lymphocyte‐associated protein 4 (CTLA‐4) as a predicted regulator in these processes. Thus, our results suggest that subclinical malaria infections are not benign and do not support the development of immune processes required for the thorough control of parasite replication. As proof of concept, we demonstrated a role for CTLA‐4 in the development of asymptomatic recrudescent parasitemia in infection models.

## Results

### Cohort characteristics

**Figure 1:**
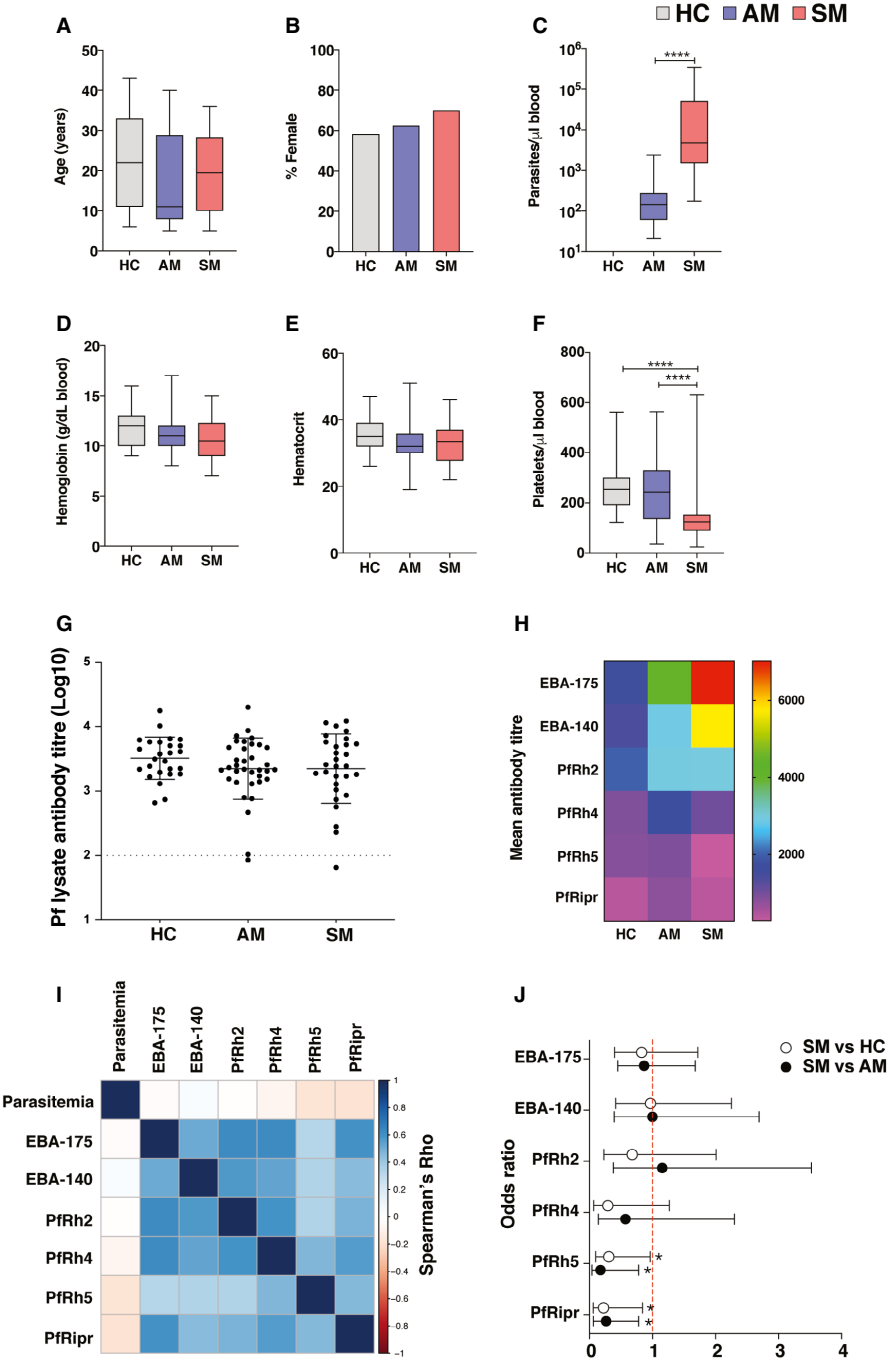
Study cohort characteristics *P. falciparum* symptomatic (*n* = 30, SM) and asymptomatic (*n* = 40, AM) infected individuals, as well as light‐microscopy and PCR parasite‐negative healthy immune controls (*n* = 31, HC) were recruited for the study.
A–FClinical parameters determined in the study include age (A), gender (B), parasitemia (C), hemoglobin (g/dl blood) (D), hematocrit (E), and platelet count (F). Boxes represent the 25^th^ to 75^th^ percentiles, whiskers show the range (minimum to maximum), and lines represent the median. Significance was determined by the Kruskal–Wallis test with Dunn’s multiple comparisons (A, D–F), the Chi‐square test (B), and the Mann–Whitney test (C) using 30 (symptomatic), 40 (asymptomatic), and 31 (healthy immune controls) biological replicates. ****P < 0.0001.GAntibody titers specific for *P*. *falciparum* parasite lysate. Bars represent the mean ± SD of using 29 (symptomatic), 35 (asymptomatic), and 26 (healthy immune controls) biological replicates. The dotted line depicts the average antibody background levels of malaria‐naive healthy Melbourne controls.HMean antibody titers as determined by ELISA to the *P. falciparum* recombinant proteins EBA‐175, EBA‐140, PfRh2, PfRh4, PfRh5, and PfRipr.ISpearman correlations between IgG antibody titers to parasite antigens and parasite density.JOdds ratios as determined by logistic regression showing the association between IgG antibody titers to recombinant proteins EBA‐175, EBA‐140, PfRh2, PfRh4, PfRh5, PfRipr, and the risk of clinical *P. falciparum* infection. Symbols represent odds ratios and lines depict the 95% confidence intervals, odds ratios below the red dashed line (1) denote reduced risk of symptomatic infection, *P < 0.05.

The study recruited symptomatic (*n* = 30) and asymptomatic (*n* = 40) *P*. *falciparum‐*infected individuals, as well as light‐microscopy and PCR parasite‐negative healthy community controls (*n* = 31). Papuans from the Timika region reside both in the lowlands where malaria exposure is common and in the highlands where malaria is absent. Migration of non‐immune adults from the highlands to lowlands means a first infection and symptomatic malaria can occur in all age groups. There were no significant differences in age or gender composition between the symptomatic, asymptomatic, and healthy controls (Fig 1A and B). As expected, mean parasitemia was higher in the symptomatic group compared with the asymptomatic group (Fig 1C). No significant differences were observed between hemoglobin or hematocrit levels (Fig 1D and E), but platelet counts were significantly lower in the symptomatic group compared with both the healthy control and asymptomatic malaria groups (Fig 1F).

Assessment of antibody responses to a *P. falciparum* parasite lysate confirmed that not only infected participants but also healthy controls had been previously exposed to *P. falciparum*, with high parasite‐specific IgG titers detected in all individuals from each group (Fig 1G). Erythrocyte‐binding antigens (EBAs) and *P. falciparum* reticulocyte binding protein‐like homologs (PfRhs), two protein families involved in invasion of the parasite into the RBC, are well‐defined targets of naturally acquired immunity to malaria (Cowman & Crabb, 2006). Total IgG levels against EBA‐175, EBA‐140, PfRh2 (a/b common region), PfRh4, PfRh5, and PfRh5‐interacting protein (PfRipr) were examined to identify associations between antibody responses and symptomatic malaria. In general, antibody titers to PfRh5 and PfRipr were lower across the cohort compared with the other invasion ligands and rarely detected among symptomatic individuals (Fig 1H). While IgG levels to EBA‐140 increased with parasite levels, antibody titers to PfRh5 and PfRipr were negatively correlated with parasitemia (Fig 1I). Furthermore, logistic regression analysis revealed that IgG levels against PfRh5 and PfRipr in asymptomatic individuals and healthy immune controls significantly reduced the odds of symptomatic infection (Fig 1J). Thus, all participants in the cohort had been exposed to *P. falciparum* malaria, and asymptomatic individuals and healthy immune controls generated circulating antibodies able to reduce the odds of symptomatic infection.

### High‐dimensional mass cytometry identifies subsets of memory B cells and T cells associated with protection from symptomatic *P. falciparum* infection

**Figure EV1:**
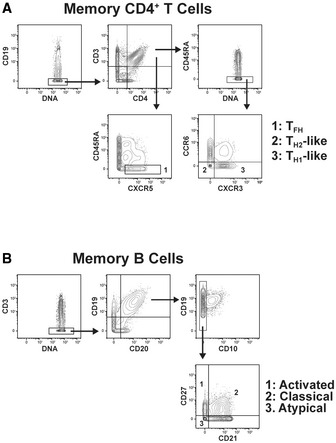
Gating strategy to define major memory CD4^+^ T cell and MBC populations PBMCs from *P. falciparum* symptomatic (*n* = 16) and asymptomatic (*n* = 24) infected individuals as well as healthy immune controls (*n* = 24) were stained with a panel of metal‐labeled antibodies and analyzed by CyTOF. Manual gating was used to select the following populations before FlowSOM clustering:
AIndividual T_H1_‐like memory CD4^+^ T cells (CD19^−^CD3^+^CD4^+^CD45RA^−^CCR6^−^CXCR3^+^), T_H2_‐like memory CD4^+^ T cells (CD19^−^CD3^+^CD4^+^CD45RA^−^CCR6^−^CXCR3^−^), circulating memory T_FH_ cells (CD19^−^CD3^+^CD4^+^CD45RA^−^CXCR5^+^) memory CD4^+^ T cells.BClassical (CD3^−^CD19^+^CD20^+^CD10^−^CD27^+^CD21^+^), atypical (CD3^−^CD19^+^CD20^+^CD10^−^CD27^−^CD21^−^), and activated MBCs (CD3^−^CD19^+^CD20^+^CD10^−^CD27^+^CD21^−^).

**Figure 2:**
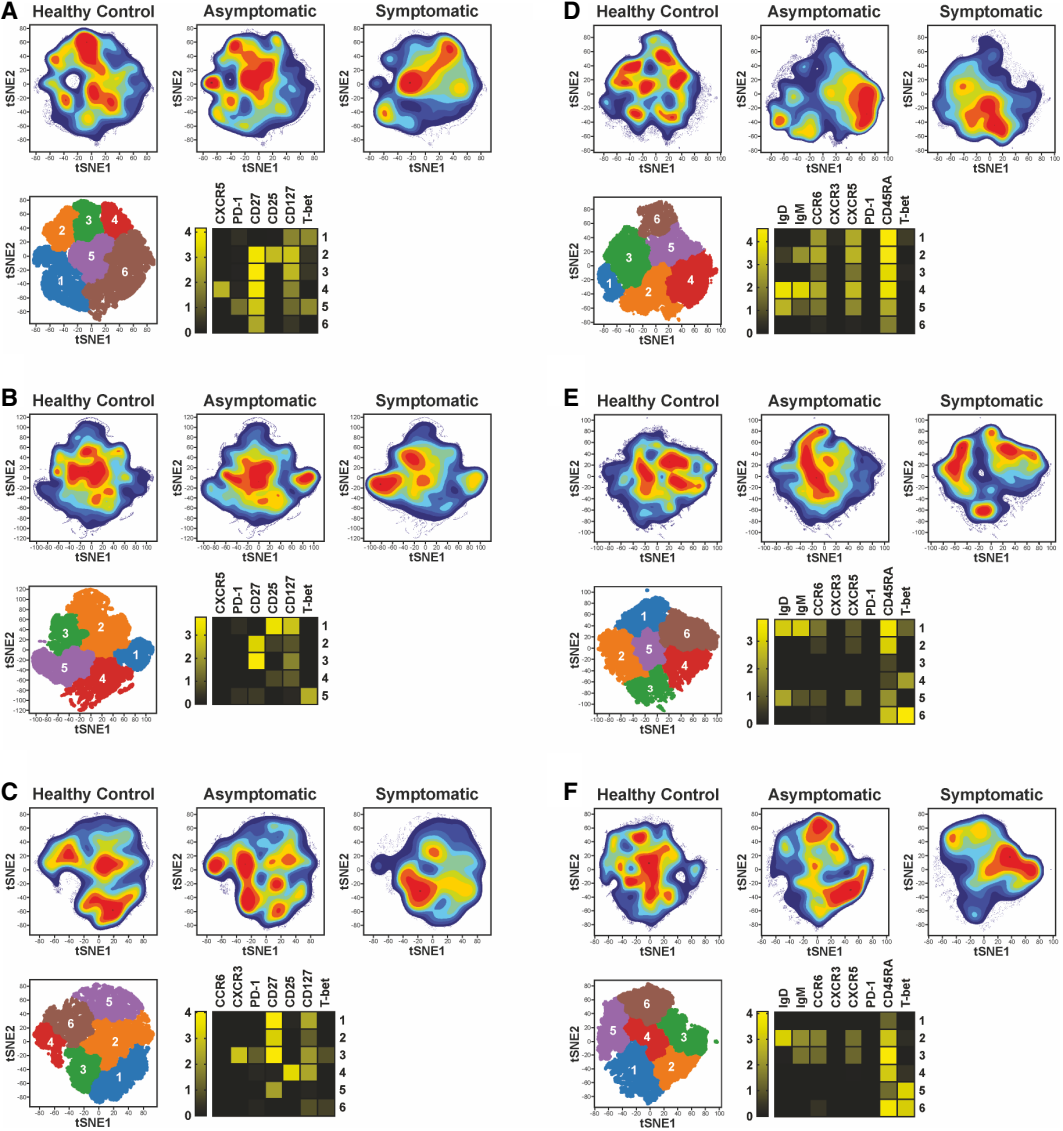
Identification of MBC and memory CD4^+^ T‐cell sub‐populations induced by *P. falciparum* symptomatic and asymptomatic infection PBMCs from *P. falciparum* symptomatic (*n* = 16) and asymptomatic (*n* = 24) infected individuals as well as healthy immune controls (*n* = 24) were stained with a panel of metal‐labelled antibodies and analyzed by CyTOF. t‐distributed Stochastic Neighbor Embedding (tSNE) analysis was performed and FlowSOM clustering was used to identify individual cell subpopulations within gated:
T_H1_‐like memory CD4^+^ T cells (CD19^−^CD3^+^CD4^+^CD45RA^−^CCR6^−^CXCR3^+^)T_H2_‐like memory CD4^+^ T cells (CD19^−^CD3^+^CD4^+^CD45RA^−^CCR6^−^CXCR3^−^)Circulating memory T_FH_ cells (CD19^−^CD3^+^CD4^+^CD45RA^−^CXCR5^+^)Classical MBCs (CD3^−^CD19^+^CD20^+^CD10^−^CD27^+^CD21^+^)Atypical MBCs (CD3^−^CD19^+^CD20^+^CD10^−^CD27^−^CD21^−^)Activated MBCs (CD3^−^CD19^+^CD20^+^CD10^−^CD27^+^CD21^−^)The tSNE plots in the top panel display cell density and represent the pooled data for each group, while the lower panel shows a projection of the FlowSOM clusters on a tSNE plot. Heatmaps show the median marker expression for each FlowSOM cluster.

To identify cellular responses associated with reduced risk of clinical *P. falciparum* malaria, PBMCs from symptomatic and asymptomatic *P. falciparum*‐infected individuals as well as healthy immune controls were stained with a panel of metal‐labeled antibodies specific for a range of B‐cell and T‐cell markers (Reagents and Tools Table) and analyzed by mass cytometry. In the memory CD4^+^ T cell pool, expression of CXCR3 and CCR6 allows the identification of T_H1_‐like CD4^+^ T cells (CXCR3^+^CCR6^−^) from T_H2_‐like CD4^+^ T cells (CXCR3^−^CCR6^−^), whereas memory T_FH_ cells may be identified by expression of CXCR5 (CD4^+^CXCR5^+^) (Fig EV1A). To reveal the cellular complexity within these pools, FlowSOM clustering was applied within each gated population. This allowed the identification of six subsets of T_H1_‐like CD4^+^ T cells, T_H2_‐like CD4^+^ T cells, and T_FH_ cells, with different expression levels of CD27, CD127, and CD25 (Fig 2A–C). Most but not all CXCR3^+^ T_H1_‐like CD4^+^ T cells expressed T‐bet (Fig 2A). Within T_H2_‐like CD4^+^ T cells, PD‐1 expression was in general low (Fig 2B). Two subsets of T‐bet^+^ T_FH_ cells were detected, as well as multiple clusters of CXCR3^−^CCR6^−^ cells within the T_FH_ pool (Fig 2C).

Within the circulating MBC pool, expression of the CD21 and CD27 markers enables the identification of CD21^+^CD27^+^ classical, CD21^−^CD27^−^ atypical, and CD21^−^CD27^+^ activated MBCs (Fig EV1B). FlowSOM clustering was performed in each population, allowing for the identification of six sub‐populations of classical, atypical, and activated MBCs (Fig 2D–F). Most classical MBC clusters expressed high levels of CD45RA, whilst expression of chemokine receptors (CXCR5 and CCR6), IgD, and IgM differed across clusters (Fig 2D). Several clusters of IgD^−^IgM^−^, class‐switched MBCs were also identified. Downstream flow cytometry experiments revealed that most of these cells expressed IgG. IgM^+^IgD^+^ and class‐switched cells were found within clusters of the three MBC sub‐populations, with populations in the atypical and activated MBC pool co‐expressing the transcription factor T‐bet (Fig 2D–F).

**Figure 3:**
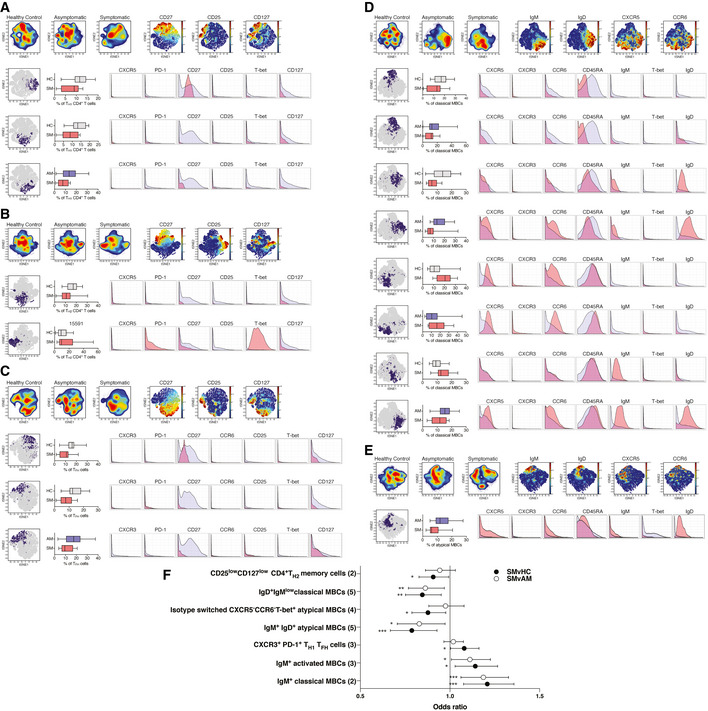
High‐dimensional mass cytometry identifies subsets of MBCs and T cells associated with reduced risk of symptomatic *P. falciparum* malaria Unsupervised identification of differentially abundant high‐dimensional cell populations between clinical groups was performed using CITRUS (FDR < 5%). between *P. falciparum* symptomatic infected individuals and asymptomatic participants or healthy immune controls.
A–EtSNE plots of clinical groups and viSNE plots depicting expression of selected surface markers, relative abundance and cellular phenotype of differentially abundant clusters identified among CXCR3^+^CCR6^−^ memory CD4^+^ T cells (A), CXCR3^−^CCR6^−^ memory CD4^+^ T cells (B), circulating memory T_FH_ cells (C), classical MBCs (D), and atypical MBCs (E). The tSNE plots in the top or each panel display cell density and represent pooled data for each group as calculated in the clustering analysis shown in Fig 2A–E, while the viSNE plots on each top panel depict relevant marker expression on tSNE overlays. The lower panels from left to right show differentially abundant populations identified in purple on a tSNE overlay, whiskers showing the range (minimum to maximum), with lines representing the median of 16 (symptomatic), 24 (asymptomatic), and 24 (healthy immune controls) biological replicates, while the pink histograms illustrate marker expression in identified differentially abundant populations, relative to background expression, shown in lilac.FOdds ratios as determined by logistic regression showing associations between cell frequencies and the risk of symptomatic *P. falciparum* infection. Symbols represent the odds ratio estimated using 16 (symptomatic, SM), 24 (asymptomatic, AM), and 24 (healthy immune controls, HC) biological replicates, and vertical lines depict the 95% confidence interval. *P < 0.05, **P < 0.01, ***P < 0.001.

Unsupervised identification of differentially abundant cell populations between clinical groups was performed using the CITRUS algorithm (FDR < 5%) (Bruggner *et al*, 2014). Populations of CD27^+^CD127^low^ T_H1_‐like CD4^+^ T cells were significantly reduced in symptomatic individuals, relative to asymptomatic counterparts (Fig 3A). In contrast, a population of CXCR3^−^ CD4^+^ T cells, expressing high T‐bet levels was significantly increased in symptomatic individuals compared to healthy immune controls (Fig 3B). Within the T_H2_‐like memory pool, a CD127^+^ cluster, expressing low levels of CD25 was enriched among healthy immune controls relative to symptomatic participants (Fig 3B). Two clusters of T_H2_‐polarized T_FH_ cells expressing CD127 and CD27 were also increased in both healthy immune controls and asymptomatic individuals compared to symptomatic counterparts (Fig 3C). CITRUS analysis revealed four main populations of classical MBCs differentially abundant between clinical groups. Whereas CXCR5^+^CCR6^+^ class‐switched and IgM^+^ MBCs were higher in symptomatic individuals, class‐switched and IgD^+^IgM^low^ MBCs expressing low CXCR5 and CCR6 levels were significantly enriched in asymptomatic participants and healthy immune controls (Fig 3D). Two subsets of IgM^+^IgD^+^ MBCs among the classical and atypical compartments were also significantly higher in asymptomatic compared to symptomatic participants (Fig 3D and E).

To define associations between cell populations and risk of symptomatic malaria, logistic regression models were applied (Benjamini–Hochberg adjusted FDR<5%). Consistent with previous studies (Obeng‐Adjei *et al*, 2015; Ryg‐Cornejo *et al*, 2016), PD‐1^+^CXCR3^+^T‐bet^+^ T_FH_ cells were associated with increased odds of *P. falciparum* symptomatic malaria (Fig 3F). Similarly, CXCR5^+^CCR6^+^ IgM^+^ classical and activated MBCs were associated with increased odds of symptomatic infection. In contrast, IgD^+^IgM^low^ classical MBCs and two populations of atypical MBCs (IgM^+^IgD^+^ and class‐switched T‐bet^+^ cells) expressing low or no CXCR5 and CCR6, along with CD127^+^CD25^low^ T_H2_‐like memory CD4^+^ T cells, were associated with reduced odds of symptomatic infection (Fig 3F). Thus, diverse MBCs expressing low chemokine receptor levels and a CD4^+^ T_H2_ cell bias were associated with reduced odds of clinical malaria.

### RNA sequencing of PBMCs segregates transcriptional profiles of symptomatic and asymptomatic *P. falciparum* malaria and healthy immune controls

**Figure EV2:**
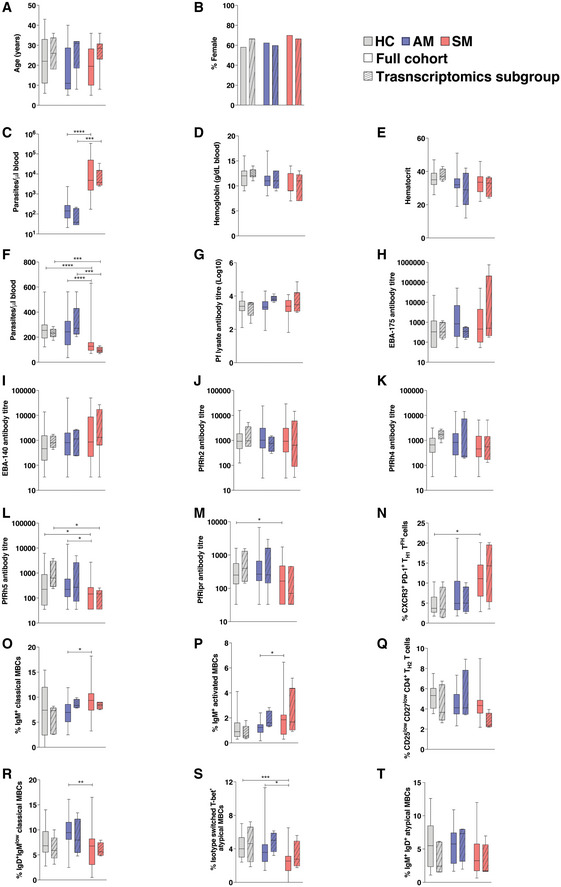
Transcriptomic cohort characteristics *P. falciparum* symptomatic (*n* = 30, SM) and asymptomatic (*n* = 40, AM) infected individuals, as well as light‐microscopy and PCR parasite‐negative healthy immune controls (*n* = 31, HC) were recruited for the study (full bars). Subsets of 5–6 samples per group were selected for PBMC transcriptional profiling (striped bars).
A–FClinical parameters determined in the study included: age (A), gender (B), parasitemia (C), hemoglobin (g/dl blood) (D), hematocrit (E), and platelet count (F).G–MAntibody responses against the following antigens were evaluated in the study which included: *P*. *falciparum* parasite lysate (G), EBA‐175 (H), EBA‐140 (I), PfRh2 (J), PfRh4 (K), PfRh5 (L), and PfRipr (M).N–TPercentage of cell populations identified by CyTOF with statistically significant odds ratios are shown: CXCR3^+^ PD‐1^+^ T_H1_ T_FH_ cells (N), IgM^+^ classical MBCs (O), IgM^+^ atypical MBCs (P), CD25^low^ CD27^low^ CD4^+^ T_H2_ memory cells (Q), IgD^+^IgM^low^ classical MBCs (R), Isotype switched T‐bet^+^ atypical MBCs (S), and IgD^+^ IgM^+^ atypical MBCs (T). Data information: Boxes represent the 25^th^ to 75^th^ percentiles, whiskers show the range (minimum to maximum), and lines represent the median. Significance was determined by the Mann–Whitney test, *P < 0.05, ****P < 0.01, ****P < 0.005, ****P < 0.001.

**Figure 4:**
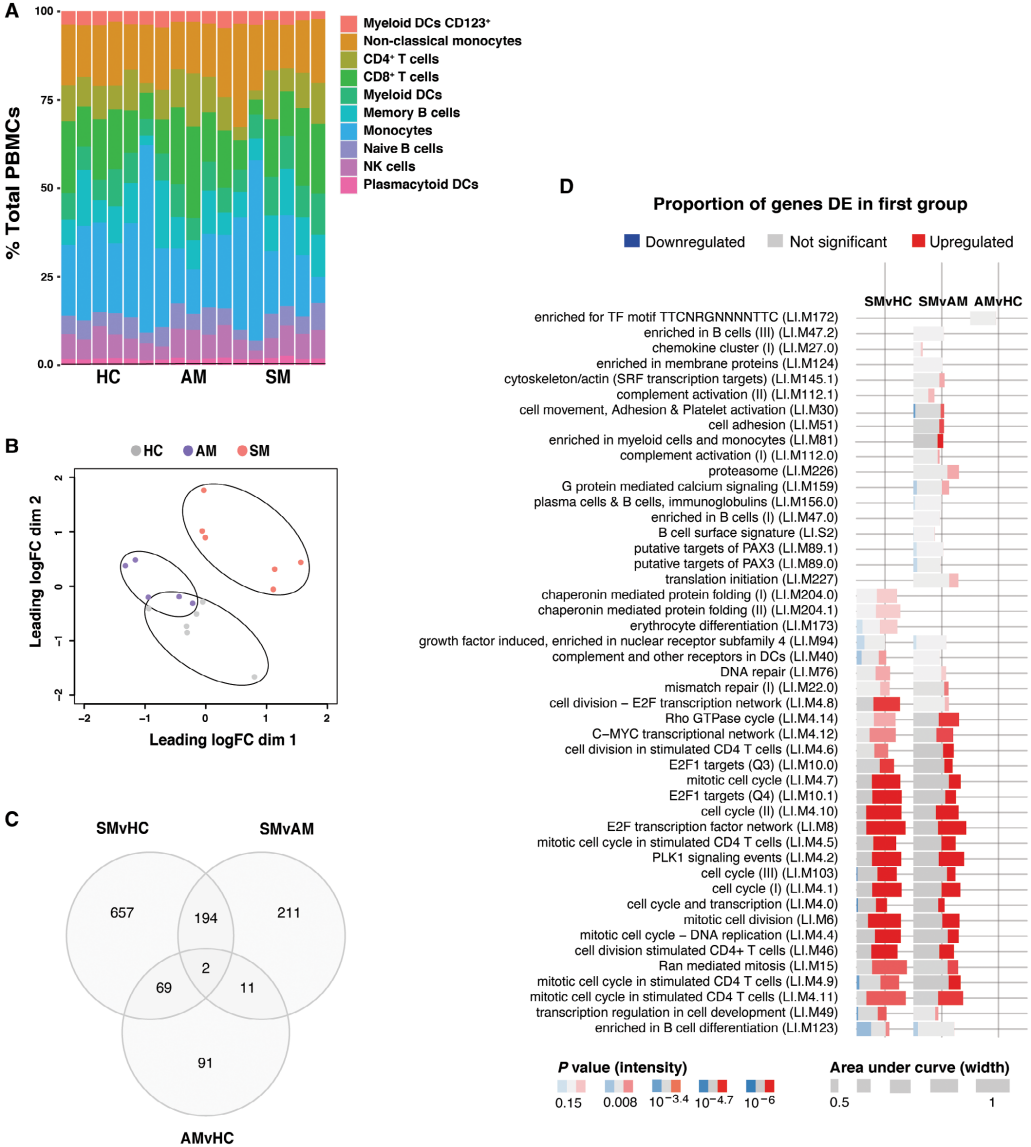
RNA sequencing of PBMCs segregates transcriptional profiles of symptomatic and asymptomatic *P. falciparum* malaria and healthy immune controls PBMCs from *P. falciparum* symptomatic (*n* = 6, SM) and asymptomatic (*n* = 5, AM) infected individuals as well as healthy immune controls (*n* = 6, HC) were selected for RNA‐seq analysis.
Estimated proportions of PBMC subpopulations determined from cell‐type deconvolution from study participant transcriptional profiles.Unsupervised multidimensional scaling of the top 1,000 most variably expressed genes across all samples.Venn diagram of the number of differentially expressed genes between clinical groups determined at a false discovery rate (FDR) of 15%Blood transcriptional module (BTM) analysis showing significant BTMs differentially enriched for pairwise comparisons (FDR<15%).

To identify molecular pathways associated with the acquisition of clinical immunity, selected *P. falciparum‐*infected symptomatic and asymptomatic study participants, as well as healthy immune controls, were chosen for transcriptional profiling by RNA‐sequencing (RNA‐seq). Clinical parameters, parasite‐specific antibody responses, and frequencies of CyTOF clusters in these samples were representative of those observed in the entire cohort (Fig EV2). As our mass cytometry analysis focused only on MBCs and CD4^+^ T cells, we used the cell‐type deconvolution package dtangle (Hunt *et al*, 2019) with hematopoietic cell RNA‐seq data (Choi *et al*, 2019) as a reference dataset to estimate the proportion of all PBMC populations in the blood (Fig 4A). No significant enrichment was observed in frequencies of total MBCs, CD4^+^ and CD8^+^ T cells, NK cells, monocyte subsets, and dendritic cells across the three clinical groups.

Multi‐dimensional scaling of transcriptional profiles revealed a good level of segregation between symptomatic *P. falciparum*‐infected individuals and asymptomatic participants, with a small degree of overlap between these individuals and healthy immune controls (Fig 4B). Clinical groups were then incorporated as a factor into linear modeling for gene expression estimation and identification of differentially expressed genes, whereby the greatest difference was found between symptomatic malaria and healthy immune controls (Fig 4C).

To identify immunological processes underlying differences in transcriptional profiles, differential enrichment was assessed with tmod (preprint: Weiner & Domaszewska, 2016) using pairwise comparisons between clinical groups and blood transcriptional modules (BTMs) (Li *et al*, 2013) as gene sets (Fig 4D). Multiple modules involved in cell division, cell development, and cell cycle were enriched in symptomatic individuals compared with both asymptomatic participants and healthy immune controls. Conversely, B‐cell differentiation and nuclear receptor subfamily 4 modules were significantly reduced in symptomatic individuals compared to asymptomatic counterparts and healthy immune controls. Interestingly, enrichment of complement, cell adhesion, and chemokine cluster modules was observed in symptomatic malaria only when compared against asymptomatic individuals, suggesting that persistent infections of low parasitemia have a regulatory effect on the induction of those pathways (Fig 4D).

### Symptomatic *P. falciparum* malaria infection drives transcriptional profiles supporting inflammatory processes and fatty acid metabolism

**Figure 5:**
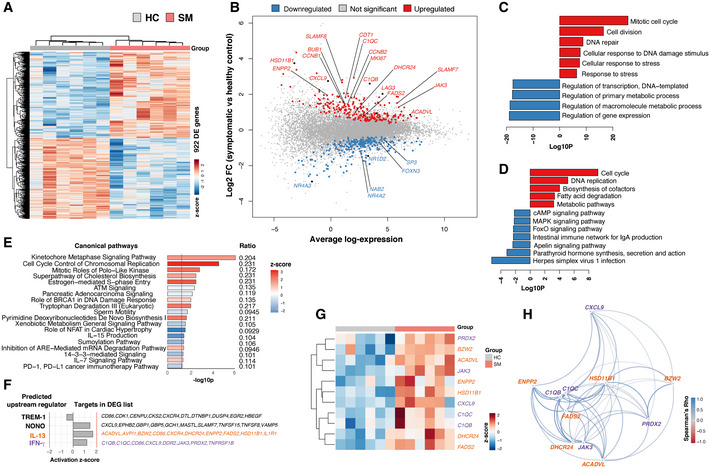
Symptomatic *P. falciparum* malaria infection drives transcriptional profiles supporting inflammatory processes and fatty acid metabolism Gene expression profiles of PBMCs from *P. falciparum* symptomatic (SM) and healthy immune controls (HC) were compared.
AUnsupervised hierarchical clustering heat map of the 922 differentially expressed genes (DEGs) between symptomatic *P. falciparum* malaria and healthy immune controls using the complete method and Euclidian measure of distance.BMean‐difference plot displaying genes differentially expressed between symptomatic *P. falciparum* malaria and healthy immune controls. Each gene is plotted as a single point determined by log‐fold‐change and average transcript abundance. Red genes are overrepresented, and blue genes are underrepresented in symptomatic malaria.CBar plots showing significantly enriched gene ontology (GO) terms scaled by Log10(*P*‐value). Red GO terms are upregulated and blue GO terms are downregulated in symptomatic *P. falciparum* malaria compared to healthy immune controls.DBar plots showing significantly enriched Kyoto Encyclopedia of Genes and Genomes (KEGG) pathways scaled by Log10(*P*‐value). Red KEGG pathways are upregulated and blue KEGG pathways are downregulated in symptomatic *P. falciparum* malaria compared to healthy immune controls.EIPA canonical pathways significantly overrepresented in DEGs between symptomatic *P. falciparum* malaria and healthy immune controls scaled by Log10(*P*‐value). Pathways with a positive *z*‐score in red are activated, and pathways with a negative *z*‐score in blue are inhibited in symptomatic *P. falciparum* malaria compared to healthy immune controls. The ratios of DEGs found in each pathway over the total number of genes in the pathway are listed on the right. The red line corresponds to a *P*‐value of 0.05.FUpstream regulator analysis of the 922 DEGs between symptomatic *P. falciparum* malaria and healthy immune controls. The red lines represent a significant activation *z*‐score of ± 2.G, HHierarchical clustering heatmap (G) and Spearman correlation network (H) displaying DEGs in symptomatic *P. falciparum* malaria and healthy immune controls predicted to be controlled by IFN‐γ and IL‐13 upstream regulators.

Unsupervised hierarchical clustering of the 922 genes differentially expressed between *P. falciparum* symptomatic participants and healthy immune controls revealed clear segregation of transcriptional profiles (Fig 5A). Gene set enrichment using gene ontology (GO) terms, Kyoto Encyclopedia of Genes and Genomes (KEGG) pathways, and Ingenuity Pathway Analysis (IPA) showed significant upregulation of terms involved in cell proliferation in symptomatic malaria (Fig 5B–E), including genes encoding cyclins such as *CCNB1* and *CCNB2*, *MKI67*, replication factors such as *CDT1* as well as mitotic checkpoint kinases like *BUB1* (Fig 5B). In addition, multiple stress response pathways were upregulated during symptomatic infection (Fig 5C–E), along with terms involved in cholesterol and fatty acid metabolism. Various genes associated with inflammatory responses were upregulated in symptomatic *P. falciparum* malaria, including *SLAMF7, SLAMF8, CXCL9, IFNG, JAK3, C1QC, C1QB*, and *LAG3* (Fig 5B).

Upstream regulator analysis identified increased activation of the transcription regulator Non‐POU domain‐containing octamer‐binding protein (NONO) and the cytokines IFN‐γ and IL‐13 in symptomatic individuals compared to healthy immune controls (Fig 5F). Whereas several genes involved in stress response and inflammatory processes were predicted as targets of NONO and IFN‐γ‐mediated pathways, the T_H2_‐cytokine IL‐13 was predicted to control genes involved in cholesterol biosynthesis and fatty acid metabolism *(ACADVL, DHCR24, ENPP2, FADS2*, and *HSD11B1)*. Interestingly, many IL‐13 and IFN‐γ‐predicted target genes upregulated in symptomatic individuals were significantly correlated with each other (Fig 5G and H), suggesting that T_H2_‐polarized responses become activated during acute malaria with the potential to help meet the energy demands posed by the inflammatory response to symptomatic infection.

Among the pathways downregulated during symptomatic infection were regulation of gene expression, metabolic processes, and signaling (Fig 5B–E). These included genes encoding DNA‐binding elements and transcription factors such as *NAB2, SP3, FOXN3*, as well as the nuclear receptors *NR4A3, NR4A2*, and *NR1D2*, known to modulate immune cell activation, cell migration, apoptosis, and cell metabolism (Fig 5B). Thus, concomitant with a proliferative/inflammatory transcriptional signature, symptomatic malaria appears to impact the control of cellular processes by altering the expression of transcriptional regulators.

### Asymptomatic *P. falciparum* malaria drives a transcriptional profile supporting immunosuppressive processes

**Figure 6:**
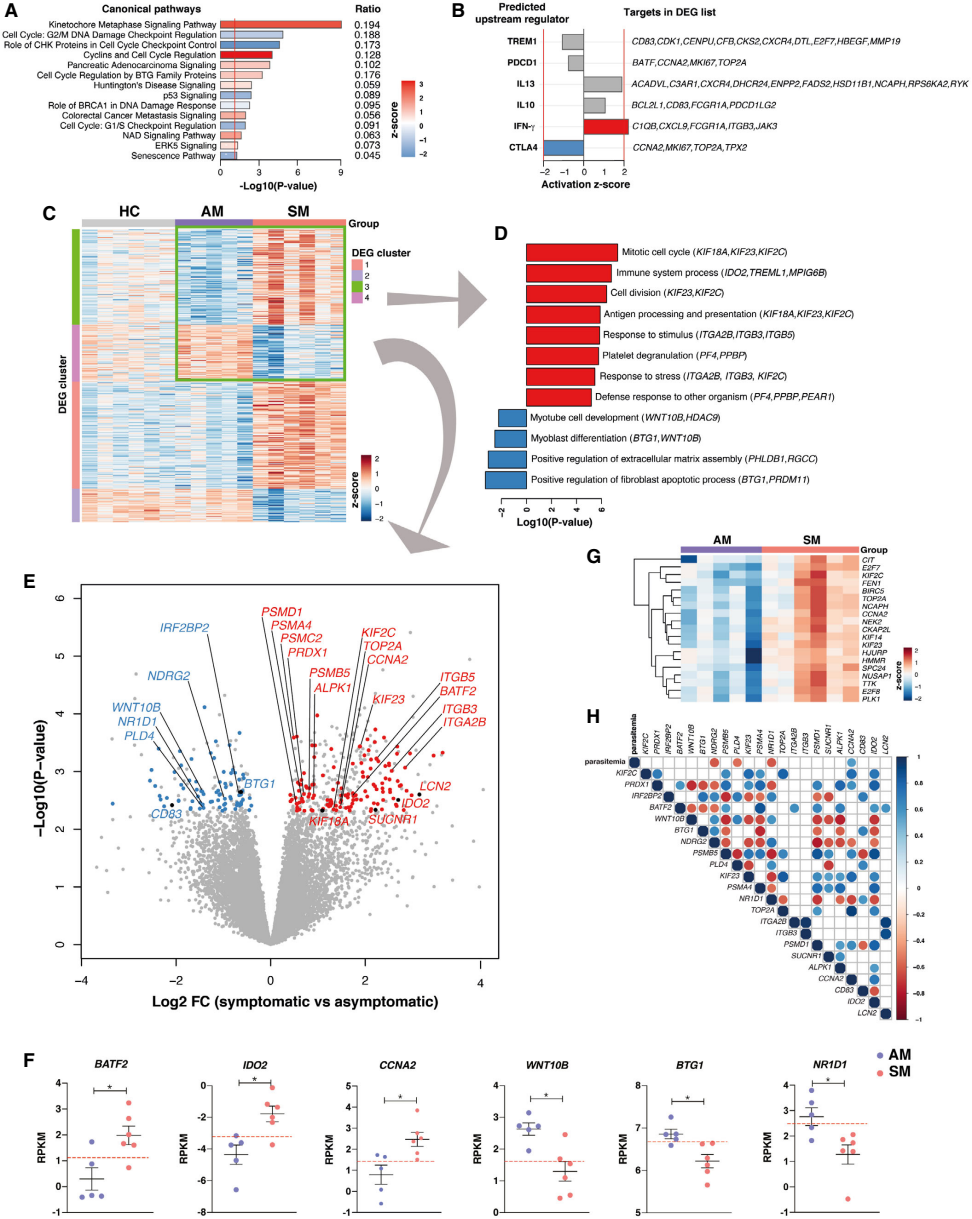
Asymptomatic *P. falciparum* malaria upregulates transcription of pathways involved in immunosuppressive processes Gene expression profiles of PBMCs from *P. falciparum* symptomatic (SM) and asymptomatic (AM) infected participants were compared.
IPA canonical pathway analysis scaled by −Log10(*P*‐value) using the 418 differentially expressed genes (DEGs) between symptomatic and asymptomatic *P. falciparum* malaria. Pathways with a positive *z*‐score in red are activated, and pathways with a negative *z*‐score in blue are inhibited in symptomatic compared with asymptomatic *P. falciparum* malaria. The ratios of DEGs found in each pathway over the total number of genes in the pathway are listed on the right. The red line corresponds to a *P*‐value of 0.05.Upstream regulator analysis of the 418 DEGs between *P. falciparum* symptomatic and asymptomatic infected participants. The red lines represent a significant activation *z*‐score of ± 2.Heatmap of the 418 DEGs in *P. falciparum* symptomatic and asymptomatic infected participants as well as in healthy immune controls.Bar plots showing significantly enriched gene ontology (GO) terms scaled by Log10(*P*‐value) in clusters 3 and 4 from the heatmap in C. Red GO terms are upregulated and blue GO terms are downregulated in symptomatic compared with asymptomatic *P. falciparum* malaria.Volcano plot displaying selected genes clusters 3 and 4 from heatmap in C scaled by Log2‐fold‐change and ‐Log10(*P*‐value) differentially expressed between *P. falciparum* symptomatic and asymptomatic infected individuals. Genes in red are overrepresented in symptomatic malaria and genes in blue are overrepresented in asymptomatic malaria.Mean Reads Per Kilobase of transcript per Million mapped reads (RPKMs) ± SEM of selected genes in *P. falciparum* malaria symptomatic (*n* = 6) and asymptomatic (*n* = 5) individuals. Dotted red lines depict transcriptional levels of healthy immune controls, Mann–Whitney test of biological replicates, *P < 0.05.Hierarchical clustering heatmap of genes associated with *CCNA2* identified by linear regression analysis (FDR < 5%).Spearman correlation matrix (Benjamini–Hochberg adjusted FDR < 5%) between parasitemia levels and immunoregulatory genes differentially expressed between symptomatic and asymptomatic *P. falciparum‐*infected individuals. Significant positive correlations are shown in blue and significant negative correlations are shown in red.

IPA of the 418 genes differentially expressed between *P. falciparum* symptomatic and asymptomatic individuals showed significant upregulation of cell proliferation canonical pathways in response to symptomatic infection (Fig 6A). In contrast, cell cycle checkpoint control, together with p53 signaling, were among the pathways downregulated in symptomatic relative to asymptomatic *P. falciparum* malaria (Fig 6A). As in Fig 5, upstream regulator analysis predicted activation of IFN‐γ and IL‐13‐mediated pathways in symptomatic malaria (Fig 6B). Notably, the only upstream regulator significantly upregulated by asymptomatic infection was CTLA‐4 (Fig 6B). All genes in the dataset predicted to be controlled by CTLA‐4 were involved in the control of cell proliferation. To further define transcriptional signatures preferentially activated by asymptomatic or symptomatic infection, hierarchical clustering was performed (Fig 6C). The inclusion of healthy immune controls in this analysis allowed visualization of 4 gene clusters. Two of them had genes either upregulated (cluster 1) or downregulated (cluster 2) in response to symptomatic infection compared to both asymptomatic infection and healthy immune controls (Fig 6C). These groups featured genes involved in response to stress, similar to those described in Fig 5. In contrast, clusters 3 and 4 consisted of genes in which expression profiles were either enriched or diminished by symptomatic or asymptomatic infection relative to homeostatic transcriptional levels from healthy immune controls (Fig 6C–F). A group of integrins (*ITGA2B, ITGB3*, and *ITGB5*), proteosome‐encoding genes (*PSMA4, PSMB5, PSMC2, PSMD1*), and cytoskeleton regulators (*KIF23, KIF2C, KIF18A*) involved in antigen presentation, along with various genes encoding protein products involved in immune defense to microorganisms (*LCN2, PRDX1, SUCNR1, BATF2, ALPK1*, and *IDO2*), were upregulated in response to symptomatic malaria but underrepresented in asymptomatic malaria carriers, suggesting that persistent asymptomatic infection may have a suppressive effect in the induction of those responses (Fig 6D–F). Similarly, genes involved in cell proliferation (*CCNA2* and *TOP2A*) predicted to be controlled by CTLA‐4 (Fig 6B) were also reduced in asymptomatic individuals (Fig 6E and F). Furthermore, linear regression analysis identified 18 additional genes involved in cell cycle control downregulated by asymptomatic malaria, with transcription patterns correlated with *CCNA2* expression (Fig 6G).

Genes in cluster 4 (Fig 6C), underrepresented in symptomatic malaria and upregulated by asymptomatic infection, included several genes encoding protein products mediating negative regulation of immune processes, such as the anti‐proliferative molecules *BTG1* and *NDRG2*, as well as the checkpoint receptor *CD83* (Fig 6D and E). *PLD4* encoding phospholipase D4 and *WNT10B* implicated in anti‐inflammatory responses (Trischler *et al*, 2016) were also upregulated in this group (Fig 6D–F). Two nuclear receptors described to negatively modulate transcriptional profiles of a range of immune responses were also overrepresented in asymptomatic individuals: *IRF2BP2* encoding the transcriptional repressor IFN regulatory factor 2 binding protein 2 that suppresses CD4^+^ T‐cell proliferation (Secca *et al*, 2016) and the nuclear receptor *NR1D1*, a circadian clock gene that controls the transcription of several inflammatory cytokines (Chang *et al*, 2019; Liu *et al*, 2020) (Fig 6E and F). Correlation analysis of this immunoregulatory signature with parasitemia levels identified two groups of genes. *NR1D1*, *NDRG2*, and *PLD4* showed significant negative correlations with parasitemia, suggesting that downregulation of transcription could be driven by high parasite burden and/or the concomitant inflammation associated with symptomatic infection (Fig 6H). In contrast, integrins, cytoskeleton regulators, and anti‐microbial genes along with *IRF2BP2, CD83, BTG1*, and *WNT10B* with anti‐proliferative and anti‐inflammatory activity were not a function of high parasitemia (Fig 6H), raising the possibility that low parasitemia asymptomatic *P. falciparum* infections activate a blood transcriptional profile that drives immunosuppressive processes.

**Figure 7:**
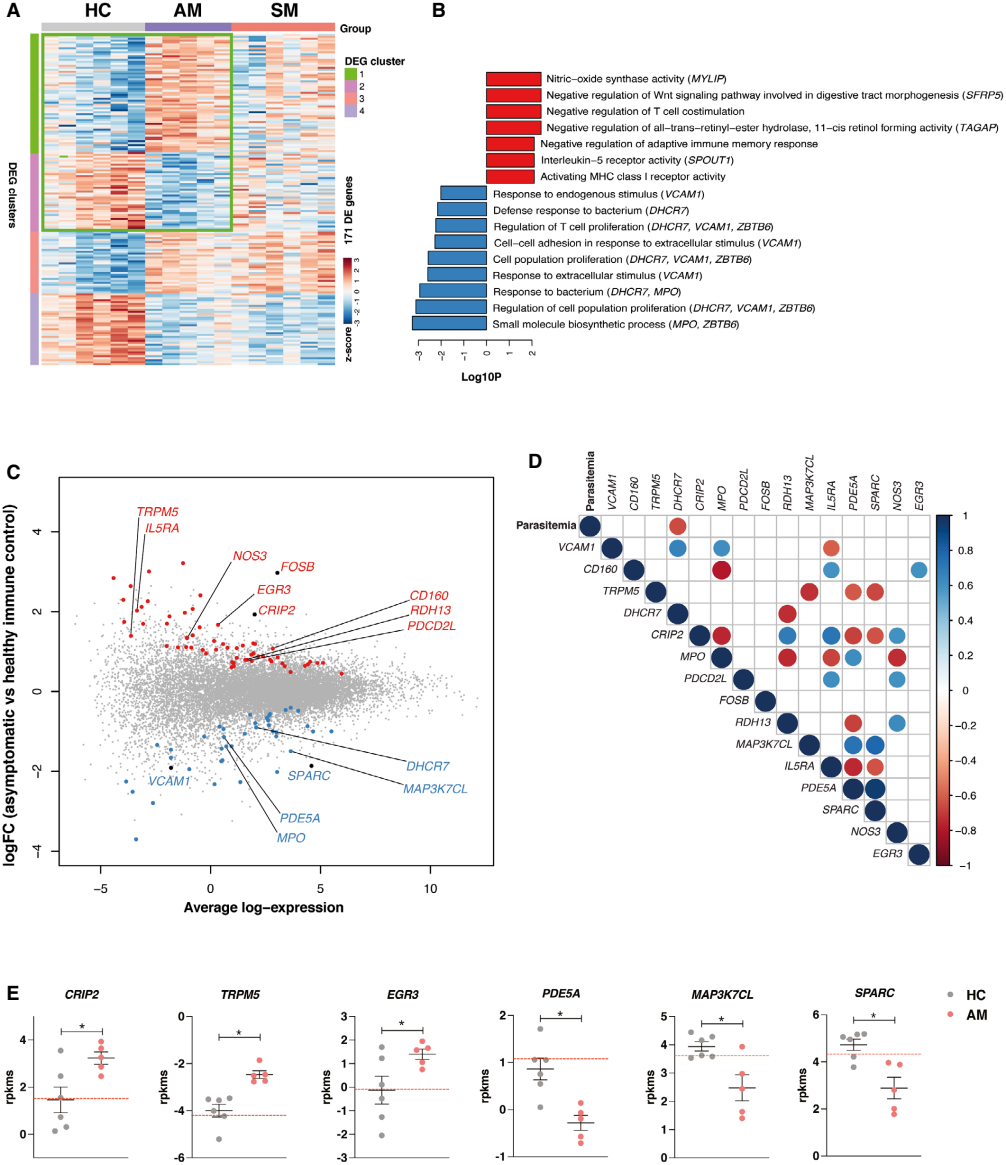
Asymptomatic *P. falciparum* malaria upregulates transcription of pathways involved in immunosuppressive processes Gene expression profiles of bulk PBMCs from *P. falciparum*‐infected asymptomatic individuals (AM) and healthy immune controls (HC) were compared.
Hierarchical clustering heat map of the 171 differentially expressed genes (DEGs) in *P. falciparum* asymptomatic and healthy immune control participants.Bar plots showing significantly enriched gene ontology (GO) terms scaled by Log10(*P*‐value) within clusters 1 and 2 from the heat map shown in A. Red GO terms are upregulated and blue GO terms are downregulated in asymptomatic *P. falciparum* malaria compared to healthy immune controls.Mean‐difference plot displaying DEGs between asymptomatic *P. falciparum* malaria and healthy immune controls. Each gene is plotted as a single point determined by log‐fold‐change and average transcript abundance. Red genes are overrepresented, and blue genes are underrepresented in asymptomatic malaria.Spearman correlation matrix (Benjamini–Hochberg adjusted FDR < 5%) between parasitemia levels and immunoregulatory genes differentially expressed between asymptomatic *P. falciparum‐*infected individuals and healthy immune controls. Significant positive correlations are shown in blue and significant negative correlations are shown in red.Mean RPKMs ± SEM of selected genes in *P. falciparum* malaria asymptomatic individuals (*n* = 5) and healthy immune controls (*n* = 6). Dotted red lines depict transcriptional levels of *P. falciparum*‐infected symptomatic individuals, Mann–Whitney test of biological replicates, *P < 0.05.

To further define molecular processes influenced by asymptomatic *P. falciparum* infection, transcriptional profiles of asymptomatic individuals and healthy immune controls were compared (Fig 7A). Within the pathways uniquely downregulated by asymptomatic malaria were response to endogenous stimulus, regulation of cell proliferation, and cell‐cell adhesion (Fig 7B). These included genes encoding 7‐dehydrocholesterol reductase (*DHCR7*) involved in vitamin D metabolism, myeloperoxidase (*MPO)* with important antimicrobial function, as well as the secreted protein acidic and rich in cysteine (*SPARC*) and vascular cell adhesion protein 1 *(VCAM1*) that play an important role in cell adhesion under inflammatory conditions. *MAP3K7CL* encoding the mitogen‐activated protein kinase 7 and *PDE5A* encoding a phosphodiesterase implicated in the control of T‐cell function were also significantly downregulated in asymptomatic individuals compared to healthy controls (Fig 7C). Gene set enrichment analysis showed significant upregulation of retinol metabolism, terms involved in negative regulation of T‐cell co‐stimulation, and nitric oxide synthesis (Fig 7B and C) in response to asymptomatic malaria. These included *RDH13*, encoding retinol dehydrogenase 13, genes involved in NFKB regulation such as *CRIP2* and *PDCD2L*, as well as *NOS3*, encoding nitric oxide synthase. The expression of *IL5RA* encoding the receptor for the T_H2_‐cytokine IL‐5 was also upregulated in asymptomatic individuals (Fig 7C). Various genes encoding protein products involved in the negative control of B cell (such as *TRPM5*) and T‐cell effector function (such as *CD160)*, as well as the transcription regulators *EGR3* (Safford *et al*, 2005) and *FOSB* (Baumann *et al*, 2003), were also upregulated in *P. falciparum* asymptomatic malaria compared to healthy individuals (Fig 7C). Except for *DHCR7*, which negatively correlated with high parasitemia, transcription profiles of immunoregulatory genes differentially expressed between asymptomatic infected individuals and healthy controls were not correlated with parasitemia levels (Fig 7D). Furthermore, various genes such as *CRIP2, TRPM5, EGR3, PDE5A, MAP3* *KTC7*, and *SPARC*, among others, displayed similar levels of upregulation or downregulation in asymptomatic individuals compared to both symptomatic counterparts and healthy controls (Fig 7E). Thus, these results support the notion that asymptomatic *P. falciparum* infections drive a blood transcriptional profile that supports anti‐inflammatory and immunosuppressive processes.

### Asymptomatic *P. falciparum* malaria‐immunosuppressive transcriptional profiles are not correlated with protective responses to infection

**Figure EV3:**
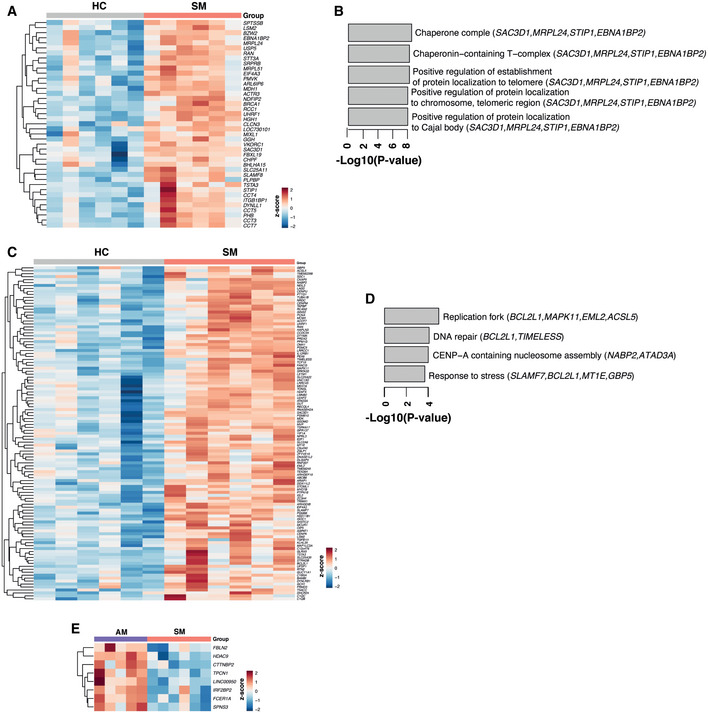
Correlations between cell populations identified by CyTOF and *P. falciparum* malaria blood transcriptional signatures A, BHierarchical clustering heatmap (A) and bar plots showing enriched GO pathways (B) using genes upregulated in symptomatic individuals that were significantly correlated (Benjamini–Hochberg adjusted *P* < 0.05) with IgM^+^ activated MBCs.C, DHierarchical clustering heatmap (B) and bar plots showing enriched GO pathways (D) using genes upregulated in symptomatic individuals that were significantly correlated with T‐bet^+^ CD4^+^ T cells.EHierarchical clustering heat map of genes upregulated in asymptomatic *P*. *falciparum‐*infected individuals that were significantly correlated (Benjamini–Hochberg adjusted *P* < 0.05) with CD27^+^ T_H2_ CD4^+^ cells.

**Figure 8:**
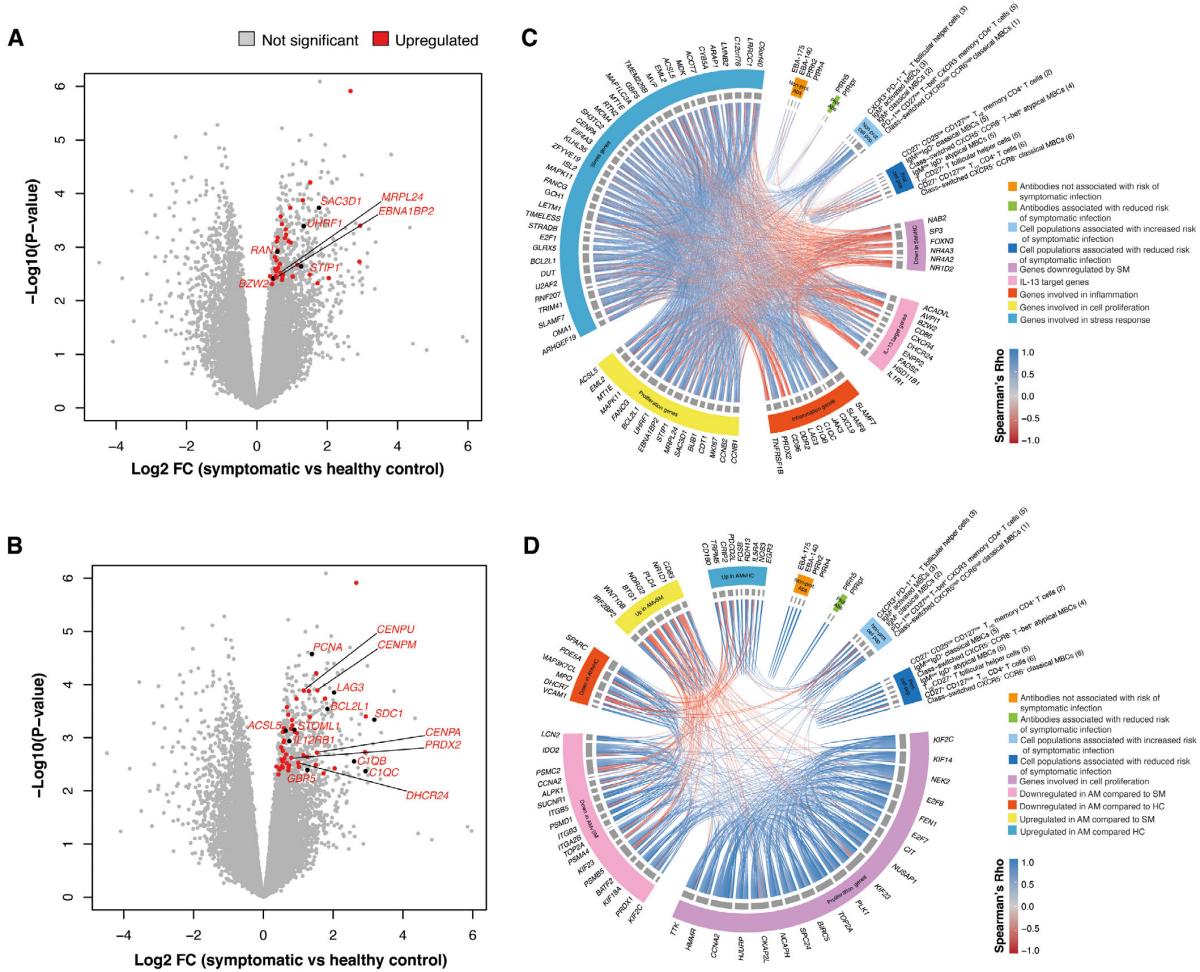
Asymptomatic *P. falciparum* malaria supports humoral responses to infection, but drives immunosuppressive responses AVolcano plot showing genes upregulated in symptomatic individuals that were significantly correlated with IgM^+^ activated MBCs.BVolcano plot showing genes upregulated in symptomatic individuals that were significantly correlated with T‐bet^+^ CD4^+^ T cells.CChord diagram integrating associations between symptomatic malaria signature genes, immune cell populations, and antibody responses. Blue lines within the chord diagram represent positive correlations between two variables, while red lines represent negative correlations (Benjamini–Hochberg adjusted Spearman’s Rho, FDR < 5%).DChord diagram integrating associations between asymptomatic malaria signature genes, immune cell populations, and antibody responses. Blue lines within the chord diagram represent positive correlations between two variables, while red lines represent negative correlations (Benjamini–Hochberg adjusted Spearman’s Rho, FDR < 5%).

**Figure EV4:**
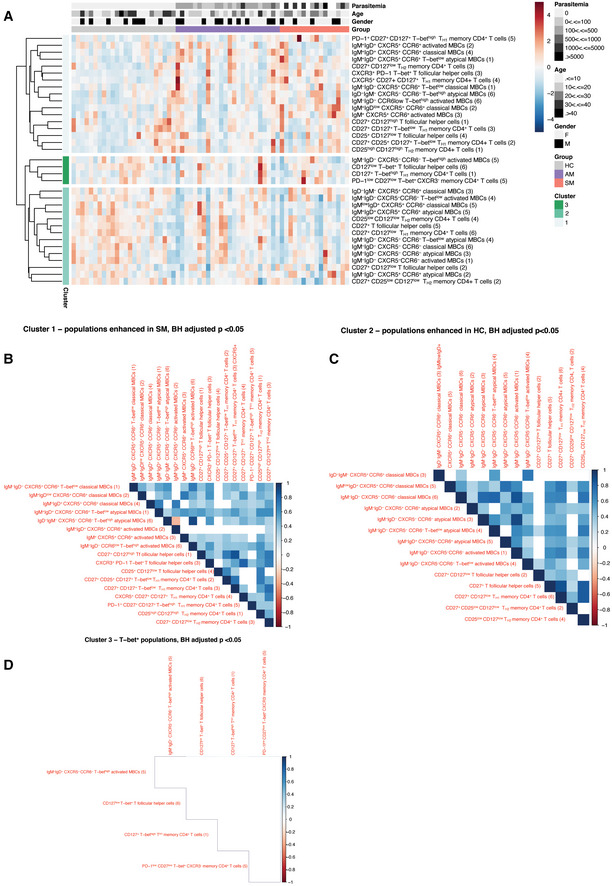
Correlations between cell populations identified by CyTOF in response to *P. falciparum* infection PBMCs from *P. falciparum* symptomatic (*n* = 16, SM) and asymptomatic (*n* = 24, AM) infected individuals as well as healthy immune controls (*n* = 24, HC) were stained with a panel of metal‐labeled antibodies and analyzed by CyTOF.
AHierarchical clustering subdivides population identified by CyTOF into three main clusters, with cluster 1 featuring populations abundant in symptomatic patients and cluster 3 cells abundant in healthy immune controls.B–DSpearman correlation matrices were used to determine the relationship between cell populations within each separate cluster (Benjamini–Hochberg adjusted *P* > 0.05). The numbers beween parentheses next to each cell population indicate the cluster number within each subset defined by FlowSOM clustering in Fig 2.

To integrate immune responses associated with increased or reduced risk of malaria identified by mass cytometry and blood transcriptional signatures, Spearman correlations were applied (Benjamini–Hochberg adjusted FDR < 5%). In general, cell populations overrepresented in symptomatic malaria were positively correlated with the symptomatic transcriptional signature, with T‐bet^+^ memory CD4^+^ T cells and activated IgM^+^ MBCs featuring the highest number of associations with gene expression profiles (Fig EV3). Whereas expression of genes involved in cell cycle progression, inflammatory and stress responses positively correlated with frequencies of T‐bet^+^ memory CD4^+^ T cells (Figs 8A and EV3A and B), genes involved in cell proliferation and telomerase recruitment were associated with the expansion of the activated IgM^+^ MBC compartment (Figs 8B and EV3C and D). Frequencies of activated IgM^+^ MBCs, associated with increased odds of symptomatic infection, were also positively correlated with classical IgM^+^ MBCs and with CXCR3^+^ T_FH_ cells (Figs 8C and EV4A and B). Together, these findings suggest that the highly inflammatory milieu during symptomatic infection supports a T_H1_‐polarized T_FH_ cell compartment, which induces activation and expansion of this MBC pool (Fig 8C).

Populations of class‐switched and IgD^+^IgM^low^ MBCs associated with reduced odds of symptomatic infection, that were overrepresented in asymptomatic individuals and healthy immune controls, were highly correlated with each other and were also positively associated with a population of CD27^+^ T_H2_‐polarized T_FH_ cells. This suggests that help from this T_FH_ cell lineage sustains the expansion of MBCs contributing to clinical immunity (Figs 8D and EV4A and C). Transcription levels of most genes enriched in asymptomatic infection were positively associated with each other (Fig 8D). Unlike symptomatic malaria, where blood transcriptional profiles showed coherent correlations with key immune cell populations expanding in response to infection, virtually no associations were found between the asymptomatic transcriptional signature and cells associated with reduced risk of clinical malaria (Figs 8D and EV3E). Thus, these results suggest that two independent processes with presumably different etiologies operate during asymptomatic malaria: an antibody‐dependent immune response that reduces the risk of symptomatic infection, and an immunoregulatory signature capable of reducing immune cell effector function.

### CTLA‐4 is upregulated in memory CD4^+^ T cells in asymptomatic malaria and contributes to the development of clinically silent parasitemia

**Figure 9:**
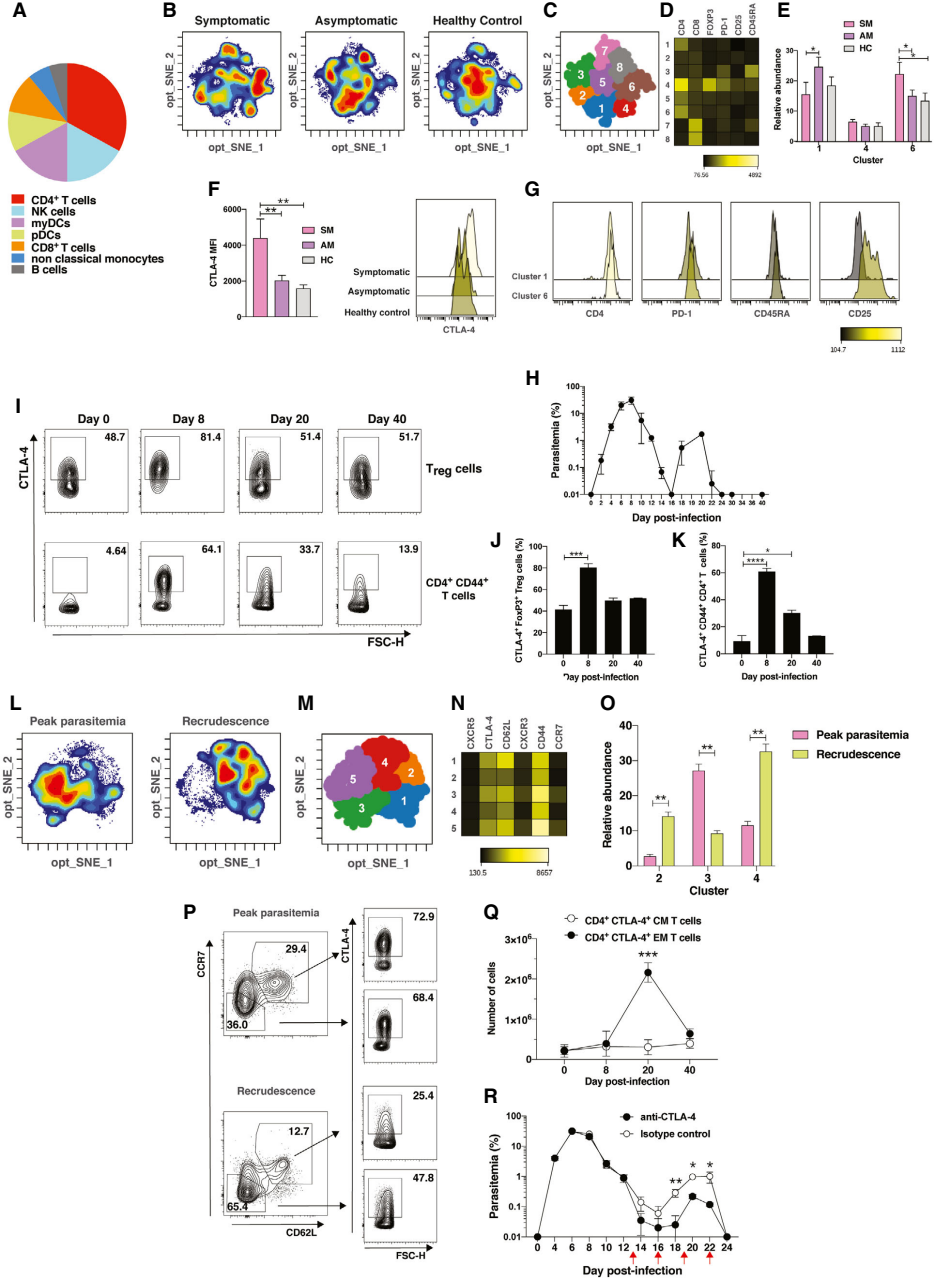
CTLA‐4 is upregulated in memory CD4^+^ T cells in asymptomatic malaria and contributes to the development of clinically silent parasitemia AEstimated proportions of PBMC subpopulations determined from cell‐type deconvolution featuring transcriptional profiles upregulated in asymptomatic individuals.B–GPBMCs from *P*. *falciparum* symptomatic (*n* = 6, SM) and asymptomatic (*n* = 6, AM) infected individuals as well as healthy immune controls (*n* = 6, HC) were stained with a panel of antibodies and analyzed by flow cytometry. tSNE plots (B) display cell density of the pooled data for each group, (C) shows a projection of the FlowSOM clusters on a tSNE plot, and (D) is a heatmap depicting the cellular phenotype of clusters of CTLA‐4^+^CD3^+^CD19^−^ cells. Bar plots representing mean relative abundance of biological replicates ± SEM of relevant clusters among clinical groups (E), Mann–Whitney test, *P < 0.05. The panel on the left shows the average CTLA‐4 mean fluorescent intensity (MFI) or 6 biological replicates in cluster 4 ± SEM, Mann–Whitney test, **P < 0.01, while representative histograms of CTLA‐4 expression in cluster 4 are shown on the right (F). Marker intensity within clusters 1 and 6 (G).HC57BL/6 mice (*n* = 10) were infected with *P*. *chabaudi chabaudi*. Percentage parasitemia of infected mice was determined every 2–3 days. Symbols represent average parasitemia ± SEM.I–QSplenocytes from *P*. *chabaudi chabaudi* infected mice were stained with fluorescent antibodies and analyzed by flow cytometry. (I) Representative contour plots showing CTLA‐4‐expressing cells among T_REG_ and CD44^+^ CD4^+^ T cells. (J, K) Percentage of CTLA‐4^+^ cells among CD4^+^FoxP3^+^ T_reg_ cells (J) and CD44^+^ activated CD4^+^ T cells (K) at peak (day 8 p.i) and recrudescent (day 20 p.i) parasitemia and after infection resolution (day 40 p.i). Bar plots represent mean percentage of 6 biological replicates ± SEM (unpaired *t*‐test, ***P < 0.005, *P < 0.05). (L–N) tSNE plots (L) display cell density of the pooled data for each group, (M) shows a projection of the FlowSOM clusters on a tSNE plot, and (N) is a heatmap depicting the cellular phenotype of CTLA‐4^+^ conventional CD4^+^ T cells during peak and recrudescent parasitemia. (O) Bar plots represent the average relative abundance of 6 biological replicates in relevant clusters ± SEM (unpaired *t*‐test, **P < 0.01). (P and Q) Representative contour plots (P) and absolute number (Q) of CTLA‐4‐expressing CD4^+^CD44^+^CCR7^+^CD62L^high^ central memory (CM) and CD4^+^CD44^+^CCR7^−^CD62L^low^ effector memory (EM) cells during peak and recrudescent parasitemia. Symbols represent the mean cell number of 6 biological replicates ± SEM (unpaired *t*‐test, ***P < 0.05).RC57BL/6 mice (*n* = 6) were infected with *P*. *chabaudi chabaudi* and treated with anti‐CTLA‐4 or isotype control every 3 days from day 13 p.i as indicated by the red arrows. Parasitemia was determined every 2–3 days. Bar plots represent mean parasitemia of biological replicates ± SEM (unpaired *t*‐test, **P < 0.01, *P < 0.05).

To infer potential cellular sources of the immunoregulatory transcriptional signature featured by asymptomatic *P. falciparum* malaria, cell‐type‐specific marker genes were examined using dtangle. This analysis predicted CD4^+^ T cells as the main sources of asymptomatic malaria transcriptional profiles (Fig 9A). We then sought to provide proof of concept for the regulatory pathways identified by our bioinformatic analysis in the control of asymptomatic infection. Since CTLA‐4 was predicted as a potential regulator in these processes (Fig 6) and is expressed by CD4^+^ T cells, this pathway was selected for further analysis. Flow cytometry followed by high‐dimensional analysis of CD3^+^CTLA‐4^+^ cells identified specific populations expressing this receptor in response to symptomatic and asymptomatic *P. falciparum* malaria. Although similar frequencies of CTLA‐4^+^CD4^+^FoxP3^+^ regulatory cells (T_REG_) were found between groups, CTLA‐4 expression in this compartment was twice as high in symptomatic compared to asymptomatic infection (Fig 9A–F). CTLA‐4^+^CD4^+^ T cells expressing high CD25 levels were also increased in symptomatic infection, but conventional CD45RA^−^ memory CD4^+^ T cells became important sources of CTLA‐4 expression during asymptomatic infection compared to symptomatic counterparts (Fig 9A–G). To extend these observations and provide mechanistic evidence for the role of CTLA‐4 in the control of asymptomatic parasitemia, C57BL/6 mice were infected with *P*. *chabaudi chabaudi*, which results in a peak of parasitemia around day 8 post‐infection (p.i) that is resolved after two weeks and followed by asymptomatic recrudescences of low parasitemia (Fig 9H). Although these recrudescences differ from persistent clinically silent infections seen in human malaria, they provide a valuable tool to investigate the role of regulatory pathways in asymptomatic parasitemia. Frequencies of CTLA‐4‐expressing cells were then examined among gated FoxP3^+^CD25^+^CD4^+^ T_REG_ cells and CD44^+^ activated CD4^+^ T cells by flow cytometry. When animals were experiencing peak parasitemia on day 8 p.i., the percentage of CTLA‐4^+^ cells significantly increased in both T_REG_ and CD4^+^ T‐cell compartments (Fig 9I–K). Whereas frequencies of CTLA‐4^+^ T_REG_ cells returned to normal levels after resolution of peak parasitemia, they remained significantly higher than uninfected controls among CD4^+^ T cells when mice were experiencing asymptomatic recrudescence on day 20 p.i (Fig 9I–K). tSNE and FlowSom analysis of gated CTLA‐4^+^CD4^+^ T cells revealed clear differences in the composition of the CTLA‐4^+^ pool expanding during peak parasitemia and asymptomatic recrudescence, with high frequencies and absolute numbers of CD44^+^CCR7^−^CD62L^low^ cells consistent with an effector memory phenotype, coinciding with the onset of recrudescent infection (Fig 9L–Q). Thus, CTLA‐4 expression remains elevated among memory CD4^+^ T cells in both human and mouse asymptomatic malaria. Furthermore, administration of anti‐CTLA‐4 blocking antibodies to *P. chabaudi chabaudi*‐infected mice from day 13 p.i resulted in a 10‐fold reduction in recrudescent parasitemia levels compared to controls (Fig 9R), providing proof of concept for CTLA‐4 as an immunoregulatory pathway contributing to the development of persistent asymptomatic parasitemia after malaria infection.

## Discussion

This study pursued a systems biology approach integrating *P. falciparum*‐specific antibody responses, multidimensional mass cytometry, and blood transcriptional profiling in adults from a malaria‐endemic area of Indonesia to understand cellular and molecular processes operating during asymptomatic *P. falciparum* infection. Aligned with the concept of clinical immunity, our findings identified antibody responses to parasite invasion ligands along with specific populations of classical and atypical MBCs associated with reduced odds of symptomatic infection in asymptomatic individuals. Notably, asymptomatic *P. falciparum* malaria was also characterized by an important immunoregulatory blood transcriptional signature with the upregulation of various pathways involved in the inhibition of CD4^+^ T‐cell function. Thus, our findings suggest that although these sub‐clinical infections allow the development of antibody responses that reduce parasitemia levels to below the threshold required to induce clinical symptoms, they might not entirely support the effective induction of cellular immune processes required for the thorough control of parasite replication.

Aligned with previous RNA‐seq analyses (Tran *et al*, 2016; Lee *et al*, 2018; Bediako *et al*, 2019) and a well‐defined role for IFN‐γ in acute malaria (Pongponratn *et al*, 2003), transcriptional profiles of *P. falciparum* symptomatic malaria predicted this cytokine as an upstream regulator of the stress response induced during infection. Concomitant with this inflammatory response, symptomatic malaria was also characterized by the upregulation of enzymes required for fatty acid metabolism. Our bioinformatic approach predicted the T_H2_ cytokine IL‐13 as the regulator responsible for these processes. Recent studies revealed that IL‐13 plays a key role in driving metabolic conditioning during endurance exercise by preserving glycogen in favor of fatty acid oxidation (Knudsen *et al*, 2020). Thus, although upregulation of type‐2 cytokines in response to malaria has been historically proposed to ameliorate the effects of excessive inflammatory responses to infection, our findings here are consistent with a model in which T_H2_ pathways might become activated during acute malaria to help meet the energy demands posed by the inflammatory response to symptomatic infection.

The high‐dimensional unsupervised single‐cell mass cytometry approach used in this study allowed us to dissect distinct T_H1_ and T_H2_‐polarized memory CD4^+^ T cells, T_FH_ cells, and specific sub‐populations of classical and atypical MBCs associated with either increased or reduced odds of *P. falciparum* symptomatic malaria. Similar to *P*. *vivax* malaria (Ioannidis *et al*, 2021), class‐switched T‐bet^+^CCR6^−^ atypical MBCs were associated with reduced odds of *P. falciparum* malaria, suggesting that specific subsets within this cell lineage, previously viewed as a predictor of poor infection outcomes (Obeng‐Adjei *et al*, 2017), might play a beneficial role in the control of parasite burden. Unlike *P*. *vivax* malaria, in which only class‐switched MBCs were associated with reduced odds of symptomatic infection (Ioannidis *et al*, 2021), populations of both classical and atypical IgD^+^IgM^+/low^ MBCs were found to be associated with protection from symptomatic *P. falciparum* infection. Parasite‐specific IgM^+^ MBCs have been detected in *P. falciparum*‐exposed individuals (Krishnamurty *et al*, 2016) and IgM antibodies specific for blood‐stage antigens have been found to be associated with protection from symptomatic malaria (Arama *et al*, 2015; Boyle *et al*, 2019). Unlike class‐switched MBCs, IgD^+^IgM^+^ MBCs have the capacity to differentiate into GC B cells upon re‐stimulation (Seifert *et al*, 2015). Consistently, mouse malaria studies found that IgM^+^ MBCs adopt a GC B‐cell phenotype upon secondary infection (Pietrzak *et al*, 2020), while sequencing studies revealed that IgM^+^ MBCs acquire further mutations upon *P. falciparum* re‐infection in children (Wendel *et al*, 2017), suggesting a role for unswitched MBCs in B‐cell receptor repertoire expansion and remodeling during malaria.

Whereas several transcriptional profiles of individuals presenting with symptomatic *P. falciparum* malaria have been successfully described (Tran *et al*, 2016; Bediako *et al*, 2019; Boldt *et al*, 2019; Nallandhighal *et al*, 2019), blood transcriptional activity induced in response to asymptomatic infection is still underreported. A longitudinal study following *P. falciparum‐*asymptomatic individuals through the dry season found transcriptional changes in several metabolic pathways (Andrade *et al*, 2020). Whole‐blood microarray analysis of children with *P. falciparum* asymptomatic malaria found chromatin remodeling‐driven gene regulation to contribute to the maintenance of an asymptomatic status (Boldt *et al*, 2019). Similarly, regulatory genes involved in chromatin remodeling were differentially expressed in monocytes from malaria‐infected men living in Burkina Faso, with lower susceptibility to *P. falciparum* malaria than sympatric tribes (Quin *et al*, 2017). Whole‐blood transcriptional profiling of children who differed in their capacity to control parasitemia and fever following *P. falciparum* infection revealed a signature of p53 activation at baseline in participants who remained asymptomatic upon re‐infection (Tran *et al*, 2019), thereby identifying a critical pathway in clinical immunity. Despite the development of protective MBCs and antibody responses in our study, transcriptional profiling revealed several integrins, proteosome‐encoding genes, and cytoskeleton regulators involved in antigen presentation underrepresented in asymptomatic malaria carriers relative to symptomatic counterparts, suggesting that persistent asymptomatic low‐parasitemia infection might have a detrimental effect in the induction of those pathways. Furthermore, our analysis identified significant downregulation of genes involved in the control of cell proliferation in asymptomatic malaria and predicted the inhibitory receptor CTLA‐4 as the upstream regulator responsible for these processes. Upregulation of CTLA‐4 in T_REG_ cells and CD4^+^ T cells has previously been shown in human (Goncalves‐Lopes *et al*, 2016; Mackroth *et al*, 2016) and mouse malaria infection models (Haque *et al*, 2010; Hafalla *et al*, 2012), and its expression has been associated with restricting pathogenic cellular responses during acute infection, at the expense of inhibiting cell proliferation (Mackroth *et al*, 2016) and antibody responses to infection (Kurup *et al*, 2017). While CTLA‐4 blockade improved control of acute parasitemia in mice, it was also found to worsen experimental cerebral malaria disease outcomes (Jacobs *et al*, 2002; Lepenies *et al*, 2007), suggesting a protective role for this receptor in the development of T cell‐mediated organ‐specific syndromes during acute infection. In all the above‐mentioned settings, upregulation of CTLA‐4 was always documented in response to acute malaria. Using human studies and mouse malaria infection models, our results revealed a novel and previously unexplored role for CTLA‐4, showing that low parasitemia levels in clinically silent persistent infections are also capable of activating this suppressive pathway, thereby preventing full control of parasite replication. Further work is required to determine if upregulation of CTLA‐4 in these settings arises to prevent pathogenic responses potentially caused by persistent low parasitemia infections.

Concomitant with the downregulation of genes promoting cell proliferation, our approach identified various transcriptional regulators with immunoregulatory and anti‐inflammatory functions arising in the blood in response to asymptomatic *P. falciparum* malaria. These included B‐cell translocation gene 1 (*BTG1*), a member of the BTG/transducer of Erb family known to inhibit cell proliferation (Rouault *et al*, 1992), and the early growth response gene 3 (*EGR3)* that has recently emerged as a regulatory molecule that is able to suppress excessive CD4^+^ T‐cell responses (Singh *et al*, 2017). Expression levels of the transcriptional repressor IFN regulatory factor 2 binding protein 2 (*IRF2BP2*), that restrains CD4^+^ T‐cell activation (Secca *et al*, 2016), were also upregulated by asymptomatic infection. More mechanistic work is now needed to examine if activation of these pathways during asymptomatic infection also modulates the control of low parasitemia infection.

Without the deployment of a highly effective vaccine, there has been an increased focus on interventions to reduce malaria transmission, including administration of artemisinin combined therapies and long‐lasting insecticide‐treated bed nets (Smithson *et al*, 2015). A central issue in the malaria eradication agenda is the challenge posed by asymptomatic infection, as this clinically silent parasite reservoir is known to perpetuate transmission (Bousema *et al*, 2004). Strategies of antimalarial mass drug administration to at‐risk populations or mass screening and treatment of asymptomatically infected individuals have been considered. However, this has raised concerns about whether these strategies will have a negative impact on the immune status of the population and increase the risk of severe malaria upon re‐infection. To date, this idea remains controversial, with epidemiological evidence showing shifts to older age in malaria‐related hospital admissions in previously eliminated areas (Ceesay *et al*, 2008), to no increased risk of clinical malaria upon re‐infection after treatment of asymptomatic infections right before the start of the wet season (Portugal *et al*, 2017). Furthermore, increasing evidence suggests that asymptomatic malaria results in detrimental effects for the host including splenomegaly, anemia, diminished learning and school performance in children, and increased incidence of invasive bacterial infections requiring hospitalization, suggesting a reduced capacity to fight infection in individuals carrying asymptomatic malaria infections (Chen *et al*, 2016). Our findings here have uncovered immunosuppression as another potentially deleterious consequence of asymptomatic malaria, which has critical implications for the administration of malaria vaccines, to populations carrying subclinical infections. In line with this concept, attenuated sporozoite vaccine formulations were found to be significantly less immunogenic in malaria‐exposed African adults than malaria‐naive adults (Ishizuka *et al*, 2016; Jongo *et al*, 2019; Sissoko *et al*, 2017), suggesting that chronic exposure to malaria limits vaccine efficacy. Thus, our results suggest that asymptomatic infections are not necessarily benign and provide a framework to consider screening and treatment of asymptomatic *P. falciparum* malaria.

## Materials and Methods

### Reagents and Tools Table

**Table:** 

Reagent or resource	Reference or source	Identifier or catalog number
**Biological samples and experimental models**		
Melbourne unexposed healthy control	Walter and Eliza Hall Institute of Medical Research Volunteer Blood Donor Registry	N/A
Timika healthy community control	Pigapu and Hirapau villages, Timika	HC
Timika asymptomatic *P. falciparum* malaria	Pigapu and Hirapau villages, Timika	AM
Timika symptomatic *P. falciparum* malaria	Rumah Sakit Mitra Masyarakat Hospital, Timika	SM
C57BL6/J (*M*. *musculus*)	Jex Laboratory, Walter and Eliza Hall Institute	N/A
**Antibodies**		
**ELISA antibodies**		
Fc‐HRP‐conjugated mouse anti‐human IgG	Southern Biotech	Clone H2; Cat# 9042‐05
**Surface marker antibodies for CYTOF**		
141Pr‐conjugated mouse anti‐human CD196/CCR6	Fluidigm	Clone 11A9; Cat# 3141014A
144Nd‐conjugated mouse anti‐human CD45RA	Fluidigm	Clone HI100; Cat# 3143006B
146Nd‐conjugated mouse anti‐human IgD	Fluidigm	Clone IA6‐2; Cat# 3146005B
147Sm‐conjugated mouse anti‐human CD20	Fluidigm	Clone 2H7; Cat# 3147001B
151Eu‐conjugated hamster anti‐human ICOS	Fluidigm	Clone DX29; Cat# 3151020B
152Sm‐conjugated mouse anti‐human CD21	Fluidigm	Clone BL13; Cat# 3152010B
153Eu‐conjugated rat anti‐human CXCR5	Fluidigm	Clone RF8B2; Cat# 3153020B
156Gd‐conjugated mouse anti‐human CXCR3	Fluidigm	Clone G025H7; Cat# 3156004B
158Gd‐conjugated mouse anti‐human CD10	Fluidigm	Clone HI10a; Cat# 3158011B
165Ho‐conjugated mouse anti‐human CD19	Fluidigm	Clone HIB19; Cat# 3165025B
167Er‐conjugated mouse anti‐human CD27	Fluidigm	Clone L128; Cat# 3167006B
170Er‐conjugated mouse anti‐human CD3	Fluidigm	Clone UCHT1; Cat# 3170001B
172Yb‐conjugated mouse anti‐human IgM	Fluidigm	Clone MHM‐88; Cat# 3172004B
174Yb‐conjugated mouse anti‐human CD4	Fluidigm	Clone SK3; Cat# 3174004B
175Lu‐conjugated mouse anti‐human PD‐1	Fluidigm	Clone EH12.2H7; Cat# 3175008B
176Yb‐conjugated mouse anti‐human CD127	Fluidigm	Clone A019D5; Cat# 3176004B
APC‐conjugated rat anti‐human IgG	Biolegend	Clone M1310G05; Cat# 410711
PE‐conjugated mouse anti‐human FcRL5	Biolegend	Clone 509f6; Cat# 340304
145Nd‐conjugated mouse anti‐human PE	Fluidigm	Clone PE001; Cat# 3145006B
162Dy‐conjugated mouse anti‐human APC	Fluidigm	Clone APC003; Cat# 3162006B
161Dy‐conjugated mouse anti‐human T‐bet	Fluidigm	Clone 4B10; Cat# 3161014B
**Infection model antibodies**		
Syrian hamster anti‐mouse CTLA‐4	Walter and Eliza Hall Institute	Clone 9H10
Ultra‐LEAF™ Purified Armenian Hamster IgG Isotype Control	Biolegend	Clone 400959; Cat# 400969
**Flow cytometry antibodies**		
AF700‐conjugated mouse anti‐human CD3	Biolegend	Clone UCHT1; Cat# 300424
BV786‐conjugated mouse anti‐human CD4	BD Biosciences	Clone SK3; Cat# 664528
BV421‐conjugated mouse anti‐human CD19	Biolegend	Clone HIB19; Cat# 302234
PE/Cy7‐conjugated mouse anti‐human CD25	Biolegend	Clone M‐A251; Cat# 356108
BV711‐conjugated mouse anti‐human PD‐1	Biolegend	Clone EH12.2H7; Cat# 329928
BV650‐conjugated mouse anti‐human CD45RA	Biolegend	Clone HI100; Cat# 304136
APC/Fire750‐conjugated mouse anti‐human CD8	Biolegend	Clone SK1; Cat# 980914
PE/Dazzle594‐conjugated mouse anti‐human CTLA‐4	Biolegend	Clone BNI3; Cat# 369616
BB700‐conjugated mouse anti‐human FoxP3	BD Biosciences	Clone 236A/E7; Cat# 566526
Rat anti‐mouse CD16/CD32 Fc Block	BD Biosciences	Clone 2.4G2; Cat# 553142
FITC‐conjugated hamster anti‐mouse TCR β chain	BD Biosciences	Clone H57‐597; Cat# 553170
PE/Cy7‐conjugated rat anti‐mouse CD4	Biolegend	Clone RM4‐5; Cat# 100528
BV785‐conjugated anti‐mouse CD44	Biolegend	Clone IM7; Cat# 103041
PerCp‐Cy5.5‐conjugated rat anti‐mouse CXCR5	BD Biosciences	Clone 2G8; Cat# 560528
BV421‐conjugated Armenian hamster anti‐mouse CXCR3	Biolegend	Clone CXCR3‐173; Cat# 126521
APC/Cy7‐conjugated rat anti‐mouse CD62L	Biolegend	Clone MEL‐14; Cat#104428
Biotin‐conjugated rat anti‐mouse CCR7	Biolegend	Clone 4B12; Cat# 120103
Pacific Blue™‐conjugated rat anti‐mouse FoxP3	Biolegend	Clone MF‐14; Cat# 126409
APC‐conjugated Armenian hamster anti‐mouse CTLA‐4	Biolegend	Clone UC10‐4B9; Cat# 106309
**Chemicals, Enzymes, and other reagents**		
*P. falciparum lysate* (5 μg/ml)	Generated in‐house	N/A
*P. falciparum* recombinant protein (0.5–2 μg/ml)	Generated in‐house	N/A
1x10^5^ *P. chabaudi chabaudi* AS	Generated in‐house	N/A
Tetramethyl‐benzidine/H_2_O_2_	KPL Inc.	Cat# 50‐76‐03
Cell‐ID Cisplatin (5 μM)	Fluidigm	Cat# 201064
Dulbecco’s phosphate‐buffered saline (DPBS)	Gibco	Cat# 14190144
Human TruStain FcX	Biolegend	Cat# 422302
Bovine serum albumin (0.5%) (w/v)	Sigma‐Aldrich	Cat# A7906‐100G
Sodium azide 0.02% (w/v)	Sigma‐Aldrich	Cat# S8032‐25G
Maxpar nuclear antigen staining buffer set	Fluidigm	Cat# 201063
Maxpar fix and perm buffer	Fluidigm	Cat# 201067
Cell‐ID intercalator (125nM)	Fluidigm	Cat# 201192A
EQ Four Element beads	Fluidigm	Cat# 201078
Fixable Viability Dye efluor‐506	eBioscience	Cat# 65‐0866
**Software**		
CyTOF software v7.0.8493	Fluidigm	N/A
FlowJo v10	BD Biosciences	https://www.graphpad.com/
Cytobank	(Kotecha *et al*, 2010)	https://www.beckman.com/flow‐cytometry/software/cytobank‐enterprise
GraphPad Prism v8.0	GraphPad	https://www.graphpad.com/
Rstudio v4.0.3‐4.1.2	(R Core Team, 2021)	https://www.R‐project.org/
Rsubread v2.0.1	(Liao *et al*, 2019) (Liao *et al*, 2013)	http://bioconductor.org/packages/release/bioc/html/Rsubread.html
limma v3.46.0	(Ritchie *et al*, 2015)	http://bioconductor.org/packages/release/bioc/html/limma.html
Ingenuity Pathway Analysis	QIAGEN	http://pages.ingenuity.com/rs/ingenuity/images/IPA_data_sheet.pdf
**Reagent kits and Instruments**		
QIAamp DNA Blood Mini Kit	QIAGEN	Cat# 51104
Chameleon plate reader	Hidex	N/A
Helios mass cytometer	Fluidigm	N/A
Isolate II RNA Mini Kit	Bioline	Cat# BIO‐52072
Illumina TruSeq RNA Library Prep Kit (< 100 ng)	Illumina	Cat# RS‐122‐2001
FoxP3/Transcription Factor Staining Buffer Set	eBioscience	Cat# 00‐5521‐00
BD LSR Fortessa X20	eBioscience	N/A
Agilent TapeStation 2200	Agilent	N/A
Illumina NextSeq 500	Illumina	N/A

### Methods and Protocols

#### Study population

A retrospective study was conducted in the Timika region of Papua, Indonesia. Papuans reside both in the Timika lowlands where malaria exposure is common and the highlands where malaria is absent (Karyana *et al*, 2008). Migration of non‐immune adults from the highlands to lowlands means a first malaria infection can occur in all age groups. Consenting participants (aged between 5 and 45 years) donated a venous blood sample at enrollment, and peripheral blood mononuclear cells (PBMCs) and plasma were frozen. Parasite densities were determined by blood smears and participants with light‐microscopy confirmed malaria infections received first‐line anti‐malarial treatment according to the Indonesian Ministry of Health guidelines. In addition, 30 individuals presenting with symptomatic *P. falciparum* malaria at the Rumah Sakit Mitra Masyarakat Hospital were enrolled in the study. Hemoglobin, hematocrit, and platelet count were measured using a hematology analyzer. Genomic DNA was extracted from dried blood spots using the QIAamp DNA Blood Mini Kit (QIAGEN). The presence of *Plasmodium* species was confirmed by a nested PCR assay as previously described (Snounou *et al*, 1993). Symptomatic malaria cases were defined as individuals with an axillary fever temperature ≥ 37.5°C, chills, malaise, headache, or vomiting at the time of examination or up to 24 h prior to the examination and the presence of a *P*. *falciparum* positive blood smear and no other cause of fever discernible by physical exam. All individuals included in the immunity study were Papuan. Symptomatic individuals included in the immunity study had > 500 parasite/μl blood, whereas individuals with a *P*. falciparum‐positive blood smear and no clinical symptoms were classified as asymptomatic infections. Healthy immune controls had a negative light‐microscopy and PCR diagnosis. Previous exposure to malaria in these individuals was confirmed by ELISA against a *P. falciparum* parasite lysate. This study was approved by the Human Research Ethics Committees of the Eijkman Institute for Molecular Biology, the Walter and Eliza Hall Institute of Medical Research, the Northern Territory Department of Health & Families, and the Menzies School of Health Research. Written informed consent was obtained from all study participants prior to their inclusion in the study. All the experiments conformed to the principles set out in the WMA Declaration of Helsinki and the Department of Health and Human Services Belmont Report.

#### ELISA

Ninety‐six well plates (Corning) were coated with 5 μg/ml of *P. falciparum* lysate or 0.5–2 μg/ml of *P. falciparum* recombinant protein in carbonate buffer pH 9.6 and incubated at 4°C overnight. After washing, plates were blocked with 5% skim milk in phosphate‐buffered saline (PBS) for 1 h at 37°C. Blocked plates were washed with 0.05% Tween‐20 in PBS and incubated with serial two‐fold dilutions of plasma for 1 h at 37°C. After washes, plates were incubated with an HRP‐conjugated mouse anti‐human IgG antibody (Southern Biotech, USA) for 1 h at 37°C. Bound antibodies were detected by reaction of tetramethylbenzidine and H_2_O_2_ (KPL Inc., USA). The reaction was stopped by the addition of 1 M H_3_PO_4_ and absorbance read at 450 nm on a CHAMELEON plate reader (Hidex). Plasma from malaria‐naive Melbourne blood donors were included as background controls. Antibody titers were calculated as the serum dilution with an optical density (OD) value higher than that observed for negative controls at a 1/100 dilution.

#### CyTOF

PBMCs (2 × 10^6^) from *P. falciparum* symptomatic and asymptomatic infected individuals as well as healthy immune controls were stained with 5 μM Cell‐ID Cisplatin (Fluidigm) in PBS (Gibco) for 5 min at room temperature. Cells were then blocked with Human TruStain FcX (Biolegend) and stained with a cocktail of surface marker antibodies (Reagents and Tools Table) in CyTOF staining buffer (PBS with 0.5% bovine serum albumin [BSA; Sigma] and 0.02% sodium azide [Sigma]) for 30 min at room temperature. After surface staining, cells were fixed and permeabilized with a Maxpar nuclear antigen staining buffer set (Fluidigm) and then stained with a 161Dy‐conjugated anti‐T‐bet (clone 4B10; Fluidigm) antibody for 45 min at room temperature. Cells were then washed twice and stored in Maxpar fix and perm buffer (Fluidigm) with 125 nM Cell‐ID iridium intercalator (Fluidigm) for a minimum of 18 h. Prior to data acquisition, cells were washed twice by centrifugation in ultrapure water then resuspended in a 1/10 dilution of 4‐Element EQ normalization beads (Fluidigm) in ultrapure water. Cells were analyzed on a Helios model mass cytometer (Fluidigm) at ~ 300 events/s. Data were normalized using the signal from 4‐Element EQ Beads (Fluidigm) as previously described (Finck *et al*, 2013). Manual gating was performed using FlowJo version 10 (BD Biosciences) to exclude doublets and dead cells before individual cell populations were selected and exported for further analysis in Cytobank (Kotecha *et al*, 2010). Individual cell populations were then visualized using viSNE (Amir *et al*, 2013), while FlowSOM (Van Gassen *et al*, 2015) was used to examine the composition of the MBC and memory CD4^+^ T cell pools. The following parameters were included in the viSNE analysis for classical, atypical, and activated MBCs: IgD, IgM, CXCR5, CXCR3, CCR6, CD45RA, PD‐1, and T‐bet. For CXCR3^−^CCR6^+^ and CXCR3^−^CCR6^+^ memory CD4^+^ T cells, the following parameters were included: CXCR5, PD‐1, CD27, CD25, CD127, and T‐bet. For T_FH_ cells, the following parameters were included: CXCR3, CCR6, PD‐1, CD27, CD25, CD127, and T‐bet. Self‐organizing maps (SOMs) were then generated for each cell population using hierarchical consensus clustering on the tSNE axes. CITRUS (Bruggner *et al*, 2014) was used to identify differentially abundant cell populations using the same parameters as the viSNE analysis at a 5% FDR. Preliminary analysis revealed that a minimum of eight samples per group was required for identification of differentially abundant populations between groups.

#### RNA preparation and sequencing

RNA was extracted from 2 × 10^5^ PBMCs from selected samples with sufficient material to allow assessment of multiple endpoints using the ISOLATE II RNA Mini Kit (Bioline) following the manufacturer’s instructions, with the final elution step repeated once and RNA eluted into 40 μl total RNase‐free water. RNA was quantified with RNA Screen Tape on the Agilent TapeStation 2200 System. Libraries were prepared with either 50 ng or 25 ng total RNA using the Illumina TruSeq RNA Library Prep Kit (< 100 ng) following manufacturers’ instructions. Products were checked for size using D1000 ScreenTape on the Agilent TapeStation 2200 system, pooled in equimolar amounts, and submitted for sequencing by paired‐end, 80 base pair reads on an Illumina NextSeq 500 platform.

#### Transcriptional analysis

Raw sequence reads in FASTQ file format were aligned to the human reference genome GRCh38/hg38 using the align() function in Bioconductor package Rsubread version 2.0.1 with default parameters (Liao *et al*, 2013, 2019). Fragments of aligned sequences overlapping NCBI RefSeq human genes (build 38.2) were quantified with featureCounts (Liao *et al*, 2014) with the Rsubread inbuilt annotation used in the quantification. Genes with no symbols, sex‐linked genes, and immunoglobulin genes were filtered out from the analysis. Hemoglobin genes were found to be highly variable and were also filtered from the analysis. Genes with 0.5 counts per million (CPM) or higher in fewer than five samples were determined as unexpressed and filtered out, leaving 15,371 genes for differential expression analysis. Counts were transformed to log2‐CPM (logCPM), precision weighted, and quantile normalized using the voom() function (Law *et al*, 2014) in the Bioconductor package limma version 3.46.0 (Ritchie *et al*, 2015). The logCPM values were then converted to fragments per kilobase of exons per million mapped reads at the log2 scale (logFPKM). A linear model was fitted to each gene and differential expression was assessed using empirical Bayes moderated *t*‐statistics (Smyth, 2004). *P*‐values were adjusted to control the global FDR‐ across all comparisons using the “global” option of the decideTests function in the limma package. An FDR cut‐off of 15% was applied for calling differentially expressed genes. Preliminary analysis indicated that ~ 6 samples per group were adequate to observe good segregation of transcriptional profiles. Entrez Gene IDs for differentially expressed genes were entered into the goana() and kegga() functions (Young *et al*, 2010) in limma to determine overrepresentation of differentially expressed genes in Gene Ontology terms. Lists of differentially expressed genes between pairwise comparisons were also entered into Ingenuity Pathway Analysis (QIAGEN) software for canonical pathway and upstream regulator analysis. Cell‐type deconvolution was performed on log‐transformed and normalized counts using dtangle version 2.0.9 (Hunt *et al*, 2019) with default parameters and human hematopoietic cell RNA‐seq expression data as a reference data set (Choi *et al*, 2019). Differential enrichment of functional immune pathways was determined using the tmodLimmaTest() function in tmod version 0.46.2 (preprint: Weiner & Domaszewska, 2016) with blood transcription modules (Li *et al*, 2013) as gene sets, and the same contrasts as in the differential expression analysis. Heatmaps were created using pheatmap version 1.0.12. Correlations were visualized in R as either matrices, networks, or chord diagrams using corrplot version 0.92, corrr version 0.4.3, or circlize version 0.4.12 (Gu *et al*, 2014), respectively.

#### Mouse infections

Eight‐ to 12‐week‐old female mice were infected intravenously (i.v.) with 1 × 10^5^ *P. chabaudi chabaudi* AS. In some experiments, mice were treated intraperitoneally (i.p) with 100 µg of anti‐CTLA‐4 antibody (9H10) or isotype control (Syrian hamster serum, Biolegend) after resolution of peak parasitemia every 3 days. Parasitemia was assessed from Giemsa‐stained smears of tail blood. Mice were housed in individually ventilated cages. All experiments were performed in compliance with the Walter & Eliza Hall Institute Animal Ethics Committee requirements.

#### Flow cytometry

Human PBMCs were blocked with Human TruStain FcX (Biolegend) and stained with a cocktail of surface marker antibodies (Reagents and Tools table) in staining buffer (PBS with 1% HI‐FBS and 2 mM ethylenediaminetetraacetic acid [EDTA]) for 30 min on ice. After surface staining, cells were fixed and permeabilized using the FoxP3/Transcription Factor Staining Buffer Set (eBioscience) and then stained with PE Dazzle‐ anti‐CTLA‐4 (BNI3) and BB700‐anti‐FoxP3 (236/E7, BD Bioscience) antibodies for 45 min at room temperature. For mouse infection studies, splenocyte suspensions were incubated with anti‐CD16/CD32 antibody in staining buffer. Cells were washed and incubated with a cocktail of surface marker antibodies (Reagents and Tools table) for 30 min at 4°C. Secondary streptavidin conjugates were added for 30 min at 4°C following sample washes. After surface staining, cells were stained with surface markers and then fixed/permeabilized using the Foxp3/Transcription Factor Staining Buffer Set (eBioscience, CA) following the manufacturer’s protocol. Antibodies to FoxP3 (MF‐14) and CTLA‐4 (UC10‐4B9) were then added for 45 min at room temperature (all antibodies are from Biolegend unless otherwise indicated in the Reagents and Tools table). In all experiments, dead cells were excluded by staining with Fixable Viability Dye efluor‐506 (eBioscience, CA) and acquired using a BD LSR Fortessa X20 (eBioscience, CA). Analysis was performed using FlowJo version 10 (BD Biosciences). Cell populations were selected and exported for further analysis in Cytobank (Kotecha *et al*, 2010).

#### Statistical analysis

Characteristics of clinical groups were compared using one‐way ANOVA with Tukey’s multiple comparisons for continuous data that were normally distributed, and the Kruskal–Wallis test for data that did not follow normal distribution. The Mann–Whitney test was used to compare paired data and the Chi‐squared test for nominal data. Logistic regression models were fitted for pairwise comparisons between groups to determine the odds ratio for antibody titers and cell populations. Hierarchical clustering heatmaps were calculated using the complete method and Euclidian distance matrix using the pheatmap package version 1.0.12 in R. Linear regression models and differential abundance of cell populations were assessed using limma and a robust empirical Bayes procedure (Phipson *et al*, 2016). When performing differential expression analysis, a 15% FDR was considered significant. The FDR was otherwise controlled to below 5% using the method of Benjamini and Hochberg. Correlations were determined using Spearman’s rank correlation coefficient in the psych package version 2.1.9 in R. Statistical analyses were performed in GraphPad Prism version 8 and R versions 4.0.3‐4.1.2.

## Author contributions

**Stephanie I Studniberg:** Formal analysis; Validation; Investigation; Visualization; Methodology; Writing—original draft; Project administration; Writing—review and editing. **Lisa J Ioannidis:** Formal analysis; Investigation; Methodology; Writing—original draft; Project administration. **Retno A S Utami:** Formal analysis; Investigation. **Leily Trianty:** Formal analysis; Investigation. **Yang Liao:** Formal analysis. **Waruni Abeysekera:** Formal analysis. **Connie S N Li‐Wai‐Suen:** Formal analysis. **Halina M Pietrzak:** Investigation; Methodology. **Jullie Healer:** Supervision; Methodology. **Agatha M Puspitasari:** Investigation. **Dwi Apriyanti:** Investigation. **Farah Coutrier:** Investigation. **Jean R Poespoprodjo:** Resources; Supervision; Project administration. **Enny Kenangalem:** Resources; Project administration. **Benediktus Andries:** Resources; Project administration. **Pak Prayoga:** Resources. **Novita Sariyanti:** Resources; Project administration. **Gordon K Smyth:** Supervision. **Alan F Cowman:** Supervision. **Ric N Price:** Supervision; Project administration. **Rintis Noviyanti:** Conceptualization; Resources; Supervision; Investigation; Project administration. **Wei Shi:** Resources; Software; Formal analysis; Supervision; Investigation; Methodology. **Alexandra L Garnham:** Software; Formal analysis; Supervision; Investigation; Visualization; Methodology. **Diana S Hansen:** Conceptualization; Formal analysis; Supervision; Funding acquisition; Investigation; Methodology; Writing—original draft; Project administration; Writing—review and editing.

In addition to the CRediT author contributions listed above, the contributions in detail are:

SIS, LJI, and DSH conceived and designed the study. AMP, DA, FC, JRP, EK, BA, PP, NS, LT, RNP, and RN conducted the field sample collection. SIS, LJI, HMP, and RASU performed the experiments. JH and AFC provided reagents. SIS, LJI, RASU, YL, WA, CSNL‐W‐S, GKS, and DSH performed the analysis. SIS, LJI, and DSH wrote the manuscript. WS, ALG, and DSH supervised the study.

## Disclosure and competing interests statement

The authors declare that they have no conflict of interest.

## Supporting information



Expanded View Figures PDF

## Data Availability

Processed bulk RNA‐seq data generated for this study are available as GEO series GSE181179 (https://www.ncbi.nlm.nih.gov/geo/query/acc.cgi?acc=GSE181179). Raw data are available upon request, subject to approval by the Walter and Eliza Hall Institutional Data Access Committee (dataaccess@wehi.edu.au) to ensure preservation of patient confidentiality.
