# Supplementary material for: Molecular profiling reveals features of clinical immunity and immunosuppression in asymptomatic P. falciparum malaria
Source: Mol Syst Biol. 2022 Apr 27;18(4):e10824. doi: 10.15252/msb.202110824 (PMC9045086; doi:10.15252/msb.202110824)
Supplement: Supplementary file 1 — Expanded View Figures PDF [file MSB-18-e10824-s001.pdf]

Expanded View Figures

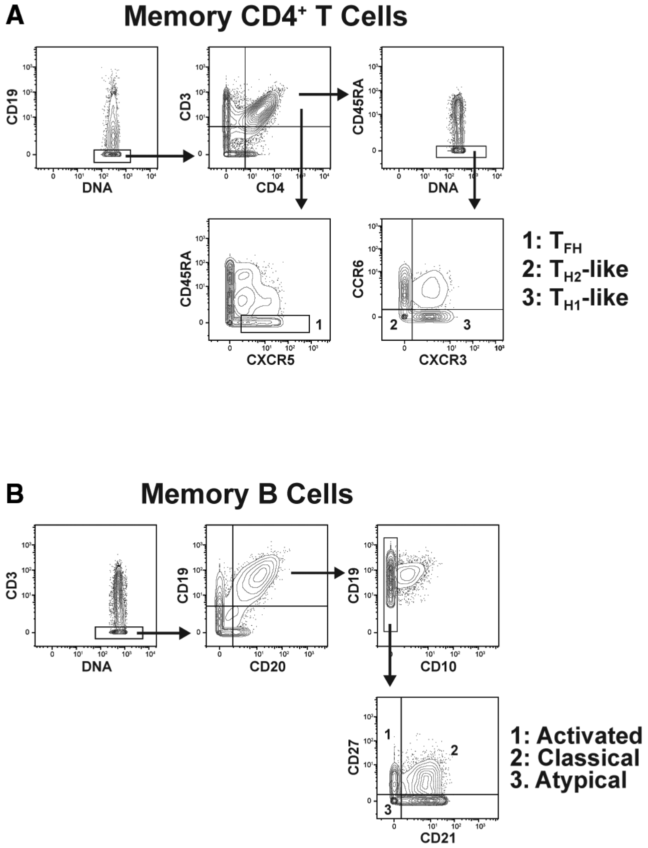

Figure EV1. Gating strategy to define major memory CD4$^+$ T cell and MBC populations.

PBMCs from *P. falciparum* symptomatic (*n* = 16) and asymptomatic (*n* = 24) infected individuals as well as healthy immune controls (*n* = 24) were stained with a panel of metal-labeled antibodies and analyzed by CyTOF. Manual gating was used to select the following populations before FlowSOM clustering:

A Individual T$_{H1}$-like memory CD4$^+$ T cells (CD19$^-$CD3$^+$CD4$^+$CD45RA$^-$CCR6$^-$CXCR3$^+$), T$_{H2}$-like memory CD4$^+$ T cells (CD19$^-$CD3$^+$CD4$^+$CD45RA$^-$CCR6$^-$CXCR3$^-$), circulating memory T$_{FH}$ cells (CD19$^-$CD3$^+$CD4$^+$CD45RA$^-$CXCR5$^+$) memory CD4$^+$ T cells.

B Classical (CD3$^-$CD19$^+$CD20$^+$CD10$^-$CD27$^+$CD21$^+$), atypical (CD3$^-$CD19$^+$CD20$^+$CD10$^-$CD27$^-$CD21$^-$), and activated MBCs (CD3$^-$CD19$^+$CD20$^+$CD10$^-$CD27$^+$CD21$^-$).

Figure EV2. Transcriptomic cohort characteristics.

P. falciparum symptomatic (*n* = 30, SM) and asymptomatic (*n* = 40, AM) infected individuals, as well as light-microscopy and PCR parasite-negative healthy immune controls (*n* = 31, HC) were recruited for the study (full bars). Subsets of 5–6 samples per group were selected for PBMC transcriptional profiling (striped bars).

A–F Clinical parameters determined in the study included: age (A), gender (B), parasitemia (C), hemoglobin (g/dl blood) (D), hematocrit (E), and platelet count (F).

G–M Antibody responses against the following antigens were evaluated in the study which included: *P. falciparum* parasite lysate (G), EBA-175 (H), EBA-140 (I), PfRh2 (J), PfRh4 (K), PfRh5 (L), and PfRipr (M).

N–T Percentage of cell populations identified by CyTOF with statistically significant odds ratios are shown: CXCR3$^+$ PD-1$^+$ T$_{H1}$ T$_{FH}$ cells (N), IgM$^+$ classical MBCs (O), IgM$^+$ atypical MBCs (P), CD25^{low} CD27^{low} CD4$^+$ T$_{H2}$ memory cells (Q), IgD$^+$IgM^{low} classical MBCs (R), Isotype switched T-bet$^+$ atypical MBCs (S), and IgD$^+$ IgM$^+$ atypical MBCs (T).

Data information: Boxes represent the 25th to 75th percentiles, whiskers show the range (minimum to maximum), and lines represent the median. Significance was determined by the Mann–Whitney test, *$P < 0.05$, ****$P < 0.01$, ****$P < 0.005$, ****$P < 0.001$.

▶

 © 2022 The Authors

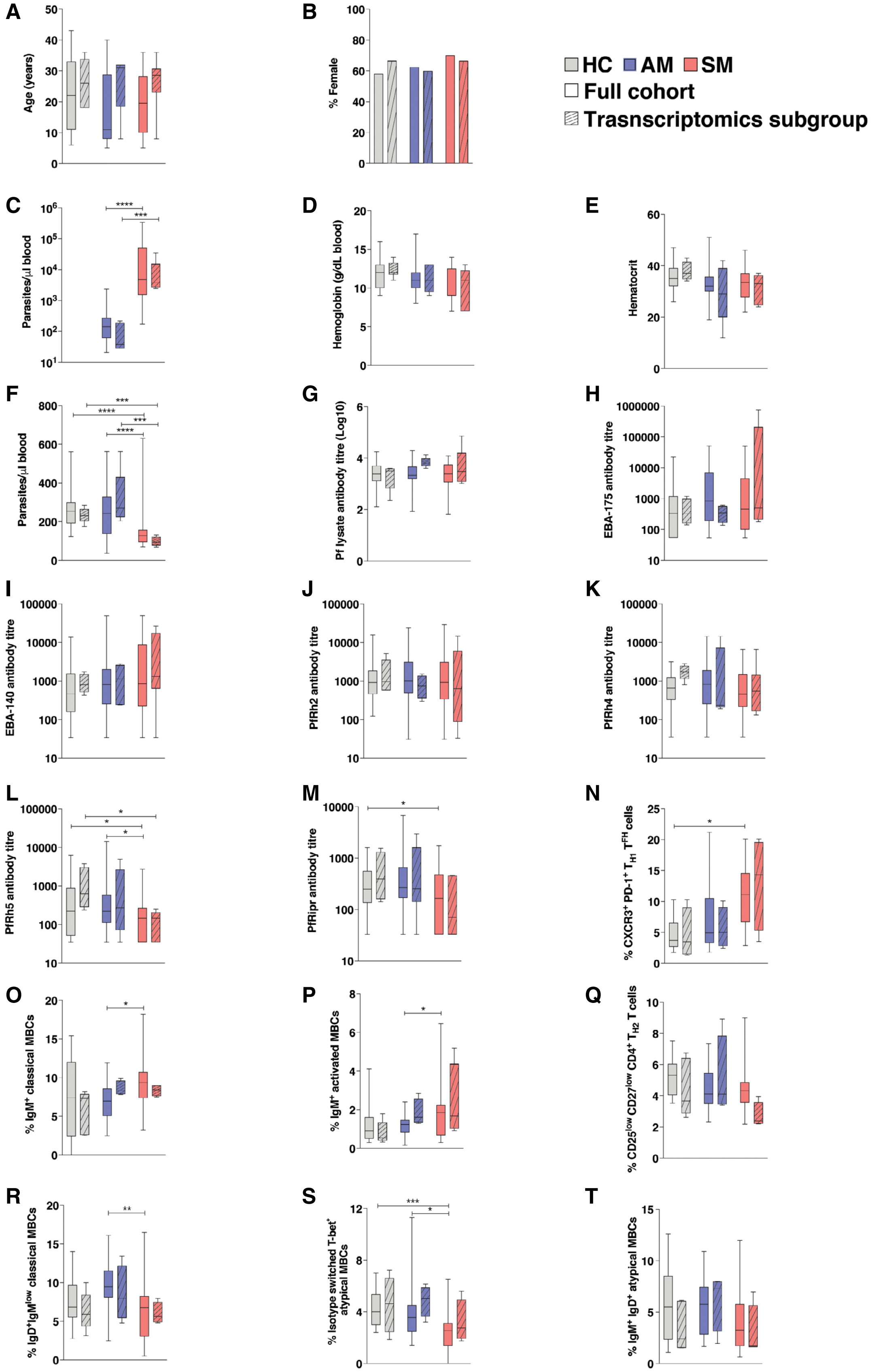
HC
AM
SM
Full cohort
Trasnscriptomics subgroup
Age (years)
% Female
Parasites/μl blood
Hemoglobin (g/dL blood)
Hematocrit
Parasites/μl blood
Pf lysate antibody titre (Log10)
EBA-175 antibody titre
EBA-140 antibody titre
PfRh2 antibody titre
PfRh4 antibody titre
PfRh5 antibody titre
PfRipr antibody titre
% CXCR3+ PD-1+ TH1 TFH cells
% IgM+ classical MBCs
% IgM+ activated MBCs
% CD25low CD27low CD4+ TH2 T cells
% IgD+IgMlow classical MBCs
% Isotype switched T-bet+ atypical MBCs
% IgM+ IgD+ atypical MBCs

Figure EV2.

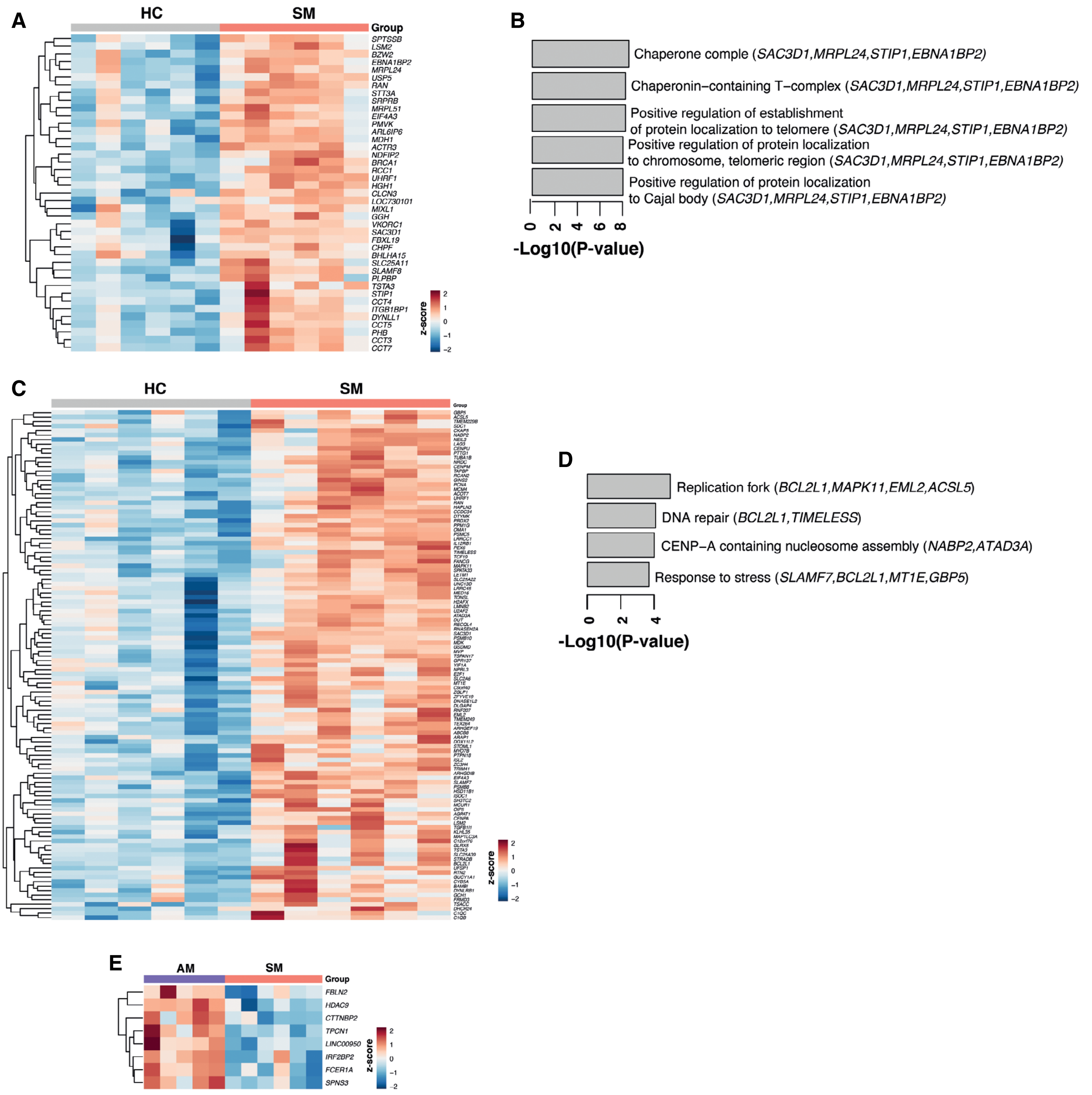

Figure EV3. Correlations between cell populations identified by CyTOF and *P. falciparum* malaria blood transcriptional signatures.

A, B Hierarchical clustering heatmap (A) and bar plots showing enriched GO pathways (B) using genes upregulated in symptomatic individuals that were significantly correlated (Benjamini–Hochberg adjusted $P < 0.05$) with IgM^+ activated MBCs.

C, D Hierarchical clustering heatmap (B) and bar plots showing enriched GO pathways (D) using genes upregulated in symptomatic individuals that were significantly correlated with T-bet^+ $CD4^+$ T cells.

E Hierarchical clustering heat map of genes upregulated in asymptomatic *P. falciparum*-infected individuals that were significantly correlated (Benjamini–Hochberg adjusted $P < 0.05$) with $CD27^+$ T_{H2} $CD4^+$ cells.

© 2022 The Authors

Figure EV4. Correlations between cell populations identified by CyTOF in response to *P. falciparum* infection.

PBMCs from *P. falciparum* symptomatic (n = 16, SM) and asymptomatic (n = 24, AM) infected individuals as well as healthy immune controls (n = 24, HC) were stained with a panel of metal-labeled antibodies and analyzed by CyTOF.

A Hierarchical clustering subdivides population identified by CyTOF into three main clusters, with cluster 1 featuring populations abundant in symptomatic patients and cluster 3 cells abundant in healthy immune controls.

B–D Spearman correlation matrices were used to determine the relationship between cell populations within each separate cluster (Benjamini–Hochberg adjusted $P > 0.05$). The numbers beween parentheses next to each cell population indicate the cluster number within each subset defined by FlowSOM clustering in Fig 2.

© 2022 The Authors

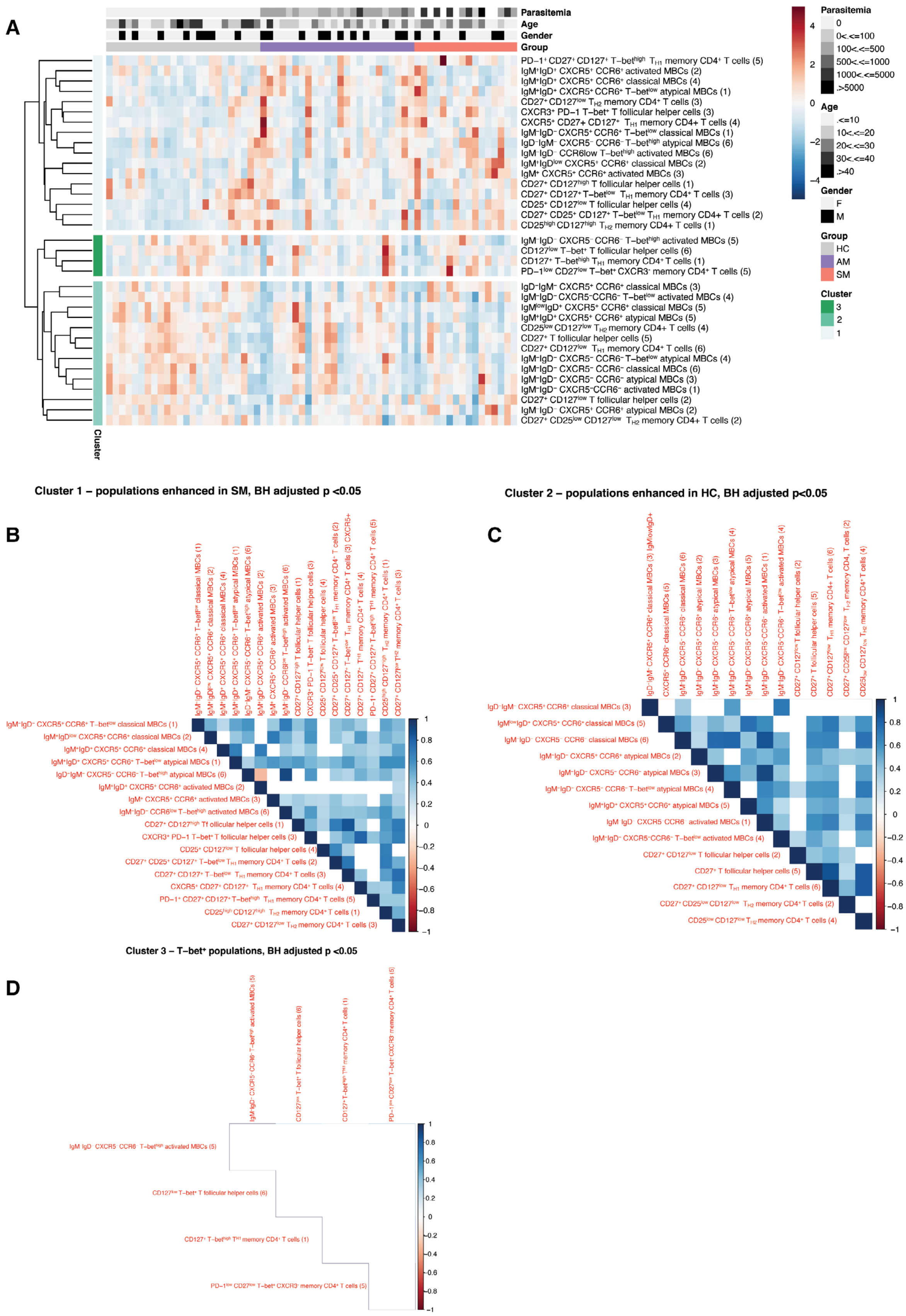

Figure EV4.

© 2022 The Authors
