# Supplementary material for: Molecular profiling reveals features of clinical immunity and immunosuppression in asymptomatic P. falciparum malaria
Source: Mol Syst Biol. 2022 Apr 27;18(4):e10824. doi: 10.15252/msb.202110824 (PMC9045086; doi:10.15252/msb.202110824)
Supplement: Supplementary file 2 [file MSB-18-e10824-s002.pdf]

molecular systems biology

Molecular profiling reveals features of clinical immunity and immunosuppression in asymptomatic P. falciparum malaria

Stephanie Studniberg, Lisa Ioannidis, Retno Utami, Leily Trianty, Yang Liao, Waruni Abeysekera, Connie Li-Wai-Suen, Halina Pietrzak, Jullie Healer, Agatha Puspitasari, Dwi Apriyanti, Farah Coutrier, Jean Poespoprodjo, Enny Kenangalem, Benediktus Andries, Pak Prayoga, Novita Sariyanti, Gordon Smyth, Alan Cowman, Ric Price, Rintis Noviyanti, Wei Shi, Alexandra Garnham, and Diana Hansen
DOI: 10.15252/msb.202110824

Corresponding author: Diana Hansen (hansen@wehi.edu.au)

Review Timeline:

Submission Date:	19th Nov 21
Editorial Decision:	7th Jan 22
Revision Received:	14th Feb 22
Editorial Decision:	22nd Mar 22
Revision Received:	30th Mar 22
Accepted:	31st Mar 22

Editor: Maria Polychronidou

Transaction Report:

(Note: With the exception of the correction of typographical or spelling errors that could be a source of ambiguity, letters and reports are not edited. Depending on transfer agreements, referee reports obtained elsewhere may or may not be included in this compilation. Referee reports are anonymous unless the Referee chooses to sign their reports.)

7th Jan 2022

Manuscript Number: MSB-2021-10824
Title: Molecular profiling reveals deleterious immunosuppression in asymptomatic P. falciparum malaria

Dear Prof. Hansen,

Thank you again for submitting your work to Molecular Systems Biology. We have now heard back from the three reviewers who agreed to evaluate your study. As you will see below, the reviewers acknowledge that the study is a relevant contribution to the field. They raise however a series of concerns, which we would ask you to address in a major revision.

I think that the reviewers' recommendations are clear and therefore there is no need to repeat the points listed below. Importantly, reviewers #2 and #3 mention that several statements indicating causality need to be better supported and/or more carefully worded to avoid overstatements. More generally, reviewers #2 and #3 recommend a more careful interpretation of the data and they make constructive suggestions on how to improve the study. All issues raised by the reviewers need to be satisfactorily addressed. Please contact me in case you would like to discuss in further detail any of the issues raised.

On a more editorial level, we would ask you to address the following points:

- Please provide a .doc file for the manuscript text (including legends for the main figures and EV Figures) and individual production-quality files for the main figures and EV Figures (one file per figure).

- Please provide 5 keywords.

- We have replaced Supplementary Information by the Expanded View (EV format). In this case, all additional figures can be included as EV Figures. EV Figures should be labeled and called out in the text as: "Figure EV1, Figure EV2... etc." Please provide one per EV Figure. EV Figure legends should be provided together with the main figure legends in the main text. For detailed instructions regarding expanded view please refer to our Author Guidelines: .

- Please provide a "standfirst text" summarizing the study in one or two sentences (approximately 250 characters), three to four "bullet points" highlighting the main findings and a "synopsis image" (550px width and max 400px height, jpeg format) to highlight the paper on our homepage.

- All Materials and Methods should be described in the main text. We would encourage you to use 'Structured Methods', our new Materials and Methods format. According to this format, the Materials and Methods section should include a Reagents and Tools Table (listing key reagents, experimental models, software and relevant equipment and including their sources and relevant identifiers) followed by a Methods and Protocols section in which we encourage the authors to describe their methods using a step-by-step protocol format with bullet points, to facilitate the adoption of the methodologies across labs. More information on how to adhere to this format as well as downloadable templates (.doc or .xls) for the Reagents and Tools Table can be found in our author guidelines:
. An example of a Method paper with Structured Methods can be found here:

- Please include a Data availability section describing how the data has been made available. This section needs to be formatted according to the example below:
The datasets and computer code produced in this study are available in the following databases:
- Chip-Seq data: Gene Expression Omnibus GSE46748 (https://www.ncbi.nlm.nih.gov/geo/query/acc.cgi?acc=GSE46748)
- Modeling computer scripts: GitHub (https://github.com/SysBioChalmers/GECKO/releases/tag/v1.0)
- [data type]: [full name of the resource] [accession number/identifier] ([doi or URL or identifiers.org/DATABASE:ACCESSION])

- For data quantification: please specify the name of the statistical test used to generate error bars and P values, the number (n) of independent experiments (specify technical or biological replicates) underlying each data point and the test used to calculate p-values in each figure legend. The figure legends should contain a basic description of n, P and the test applied. Graphs must include a description of the bars and the error bars (s.d., s.e.m.).

- The Reference list should be formatted according to the Molecular Systems Biology reference style.

- Please note that our editorial policy does not allow "Data not shown" statements.

- When you resubmit your manuscript, please download our CHECKLIST (https://bit.ly/EMBOPressAuthorChecklist) and include the completed form in your submission.
Please note that the Author Checklist will be published alongside the paper as part of the transparent process

(https://www.embopress.org/page/journal/17444292/authorguide#transparentprocess).

If you feel you can satisfactorily deal with these points and those listed by the referees, you may wish to submit a revised version of your manuscript. Please attach a covering letter giving details of the way in which you have handled each of the points raised by the referees. A revised manuscript will be once again subject to review and you probably understand that we can give you no guarantee at this stage that the eventual outcome will be favorable.

Yours sincerely,

Maria Polychronidou, PhD
Senior Editor
Molecular Systems Biology

We realize that it is difficult to revise to a specific deadline. In the interest of protecting the conceptual advance provided by the work, we recommend a revision within 3 months (7th Apr 2022). Please discuss the revision progress ahead of this time with the editor if you require more time to complete the revisions. Use the link below to submit your revision:

https://msb.msubmit.net/cgi-bin/main.plex

IMPORTANT: When you send your revision, we will require the following items:
1. the manuscript text in LaTeX, RTF or MS Word format
2. a letter with a detailed description of the changes made in response to the referees. Please specify clearly the exact places in the text (pages and paragraphs) where each change has been made in response to each specific comment given
3. three to four 'bullet points' highlighting the main findings of your study
4. a short 'blurb' text summarizing in two sentences the study (max. 250 characters)
5. a 'thumbnail image' (550px width and max 400px height, Illustrator, PowerPoint or jpeg format), which can be used as 'visual title' for the synopsis section of your paper.
6. Please include an author contributions statement after the Acknowledgements section (see https://www.embopress.org/page/journal/17444292/authorguide)
7. Please complete the CHECKLIST available at (https://bit.ly/EMBOPressAuthorChecklist).
Please note that the Author Checklist will be published alongside the paper as part of the transparent process (https://www.embopress.org/page/journal/17444292/authorguide#transparentprocess).
8. When assembling figures, please refer to our figure preparation guideline in order to ensure proper formatting and readability in print as well as on screen:
https://bit.ly/EMBOPressFigurePreparationGuideline
See also figure legend guidelines: https://www.embopress.org/page/journal/17444292/authorguide#figureformat
9. Please note that corresponding authors are required to supply an ORCID ID for their name upon submission of a revised manuscript (EMBO Press signed a joint statement to encourage ORCID adoption).
(https://www.embopress.org/page/journal/17444292/authorguide#editorialprocess)
Currently, our records indicate that the ORCID for your account is 0000-0003-4511-7918.

Please click the link below to modify this ORCID:
Link Not Available

The system will prompt you to fill in your funding and payment information. This will allow Wiley to send you a quote for the article processing charge (APC) in case of acceptance. This quote takes into account any reduction or fee waivers that you may be eligible for. Authors do not need to pay any fees before their manuscript is accepted and transferred to the publisher.

As a matter of course, please make sure that you have correctly followed the instructions for authors as given on the submission website.

*** PLEASE NOTE *** As part of the EMBO Press transparent editorial process initiative (see our Editorial at https://dx.doi.org/10.1038/msb.2010.72), Molecular Systems Biology publishes online a Review Process File with each accepted manuscripts. This file will be published in conjunction with your paper and will include the anonymous referee reports, your point-by-point response and all pertinent correspondence relating to the manuscript. If you do NOT want this File to be published, please inform the editorial office at msb@embo.org within 14 days upon receipt of the present letter.

Reviewer #1:

Studniberg et al. used a combination of systems biology tools and analysis to characterize differences among healthy individuals, symptomatic malaria, and asymptomatic malaria and found several parameters that correlate with disease severity. In addition, they discovered an immunosuppressive transcriptional signature with upregulation of pathways involved in the inhibition of T cell function, and CTLA-4. They then further validated this finding in mouse model of malaria. This is a well-designed and executed study and the manuscript is well written as well. I only have some minor questions and suggestions.
1. For Figure 1G, they need to include or discuss the Pf antibody titer in normal healthy individuals, not living in those endemic area.
2. Their PD-1 cytof signal is low. Therefore, I think it is necessary to show that the antibody itself is working.
3. Figure 3B missing label for significance.
4. I think it would be interesting to discuss a literature survey on whether people with asymptomatic malaria would have a poor response rate to regular vaccines given that they have a immunosuppressive signature.

Reviewer #2:

This is an interesting paper that contains a goldmine of data and hints at some novel inflammatory, metabolic and immune pathways associated with malaria infection. However, in its current format it presents a somewhat confusing, and at times contradictory, story. However, with a major rewrite, and perhaps a reframing of the underlying hypotheses, it could make a valuable contribution to the literature.
Firstly, the title is somewhat misleading and fails to capture the full extent of the observations presented. One aspect of the data (relating to AM) seems to have been plucked out of the many observations and highlighted, over emphasising one aspect of the data at the expense of the rest.
The focus, in the introduction and discussion, is on the potential deleterious consequences of asymptomatic malaria infections. The appropriate comparator group for such analysis is with the healthy controls, as any intervention would aim to convert these people to a healthy state, however in many of the analyses the primary comparisons are between AM and SM or between SM and HC, with much less attention paid to the AM/HC comparison. Indeed, the comparison of RNAseq data between AM and HC is only presented in the supplementary figures, not in the main text. I would argue that there are three valid comparisons to be made in this analysis: AM vs HC, AM vs SM and SM vs HC. All three should be shown wherever possible. Comparison of AM with HC is essential to determine whether AM is deleterious.
Throughout the paper, the authors refer to pathways that down regulate T cell effector functions as "immunosuppressive". Another way to think of it is that these responses are "immunoregulatory" and thus supportive of homeostasis, rather than "immunosuppressive" which implies that they are in some way pathological. The authors have not really presented any data to demonstrate that down regulation of the T cell response is detrimental.
On a related point, there is some rather simplistic thinking . For example, the implication that upregulation of inflammatory pathways that mediate disease symptoms will automatically lead to inhibition of acquisition of antibody-mediated immunity. This assumes that the concentrations of cytokines mediating inflammation are the same as those that modulate antibody responses. This is not necessarily the case and it is at least plausible that one outcome can occur independently of the other depending on the timing, concentration and wider environment of the response.
This cross-sectional study design does not allow causality to be ascribed to any of the observations and certainly does not allow the direction of any supposed causal relationship to be determined. In order to begin to demonstrate causality, and to predict which parameter predicts which, you need a longitudinal study design and/or corroboration of the observational data with experimental data. The corroboration of the CTLA-4 data in the mouse model is a very nice example of this, but it is the only example.
The manuscript is littered with comments suggesting causality and the direction of causality, e.g :
Page 8, line 7: "PD-1 +CXCR3+ T-bet+ TFH cells predicted increased susceptibility to P. falciparum symptomatic malaria
P8 line 9: "classical and activated MBCs were associated with increased risk of symptomatic infection
Page 8 Line 13 "associated with protection from symptomatic infection .....Thus, diverse MBCs expressing low chemokine receptors levels and a CD4+ TH2 cell bias predict reduced risk of clinical malaria.
Page 12. Line 8 "downregulated by asymptomatic malaria"
P12, Line 21 "indicate that low parasitemia asymptomatic P. falciparum infections activate a blood transcriptional profile that drives several immunosuppressive processes" (This could be rephrased as a hypothesis rather than stated as a fact).
Page 19 line 12 and 14; upregulated by asymptomatic infection"; "asymptomatic malaria induced the upregulation of NR1D1 "
There are many more. The entire ms. needs to be carefully checked and these statements of association or prediction removed or qualified.

I have some concerns over the study design that limit the utility of the data. No consideration is given to these limitations in either the presentation or interpretation of the data. For example,
• this is an opportunistic study based on stored samples that were not collected with this purpose in mind. To what extent has this hampered the execution of the study? Were there limitations in cell numbers, cell quality (the cells have been stored for more than a decade, how were they quality controlled?), RNA quality etc that have limited the number of assays performed.
• only a subset of samples were analysed by RNA seq. How were these samples selected? On the basis of sample quality? If so, how comparable are the groups included in the RNA seq analysis (data for the full cohort is shown in Fig 1; we need to see the same data just for the subset included in the RNA seq analysis).

• whilst the groups are roughly comparable, overall, the age range is very broad. Have you checked to see whether there are any age effects on any of the analyses?
• similarly, the range of parasitemias is quite high (and overlapping) between AM and SM. Have the data been checked for any association with parasitemia?
Fig 3 (and elsewhere) - why are all 3 groups not shown side by side on the same bar charts? Difference between AM and HC is as important as differences between AM vs SM.
Throughout the manuscript, differences are reported as being statistically significant but the statistical data are not provided. Stats should be given (in the text or figures) for all differences that are claimed to be significant, and should be adjusted for age and parasitemia if necessary.
Finally, the authors present some interesting and plausible hypotheses relating to upregulation and down regulation of different pathways. It would be really helpful - and would greatly increase the accessibility of the findings - if these ideas could be summarised in a graphical summary or graphical abstract.

Reviewer #3:

Studniberg and colleagues investigate immune phenotypes of healthy uninfected individuals, and P. falciparum infected individuals with and without malaria symptoms by mass cytometry and RNAseq and antibody responses, and search for associations. While the work is well done a presented I provide a few ideas, that I believe will help improve the manuscript.

The authors often refer to cell populations predicting increased or decreased susceptibility to P. falciparum symptomatic malaria, but their data is associative only. Individuals showing symptoms or not at the time of cell collection only, without any longitudinal data. Although interesting, i believe the wording should be revised. For instance MBCs expressing low chemokine receptors were more commonly seed in individuals without clinical malaria, but they might not be predictive of protection, and only be more expressed once symptoms arise.

I believe comparisons shown in Fig 3 would gain from showing all 3 population and not only two, it is not clear why some show HC and SM, while other show AM and SM.

How was the number samples used for PBMC RNAseq decided and how were these selected from the larger pool? this should be clear in the methods or result section.
How did the 5 or 6 samples included compare to the rest in terms of parasitemia and T and B cell subpopulations. Fig 3 could easily show the proportions of the different CD4 and MBC subpopulations and then identify the 5 or 6 samples use din Fig 4. this would also allow better discussing if RNAseq differences observed in Fig 4are likely to be promoted by different proportions of cell types, and support or not the statement "suggesting that transcriptional differences detected downstream would reflect changes in cell activity between clinical groups rather than fluctuations in the composition of the PBMC pool." which reads too strong to me without more data rot back it.
Furthermore, up-regulation of terms involved in cell proliferation in symptomatic malaria by the RNAseq data might point to increase of particular subpopulations

Why are the FDRs in Fig 4 C and D different? How many DEG would be founf if FDR would would 5% and not 15 in Fig 4C.

The authors could try to identity the cell populations/pathways promoting the gene expression differences between AM and SM, but should be cautious and have into account if gene expression differences between AM and HC are present (as in cluster 4 of Fig 6c, otherwise differences between AM and SM might be just a product of differences between symptoms or not. Accordingly, in my view some of Fig EV2 should be made available in the main manuscript figures.

In my view the authors should be caustics as to define direction and causation of the association seen in these data.

I recommend altering the title of section "Asymptomatic P. falciparum malaria supports humoral responses to infection while driving cell mediated immunosuppressive responses" and very little if anything on the data shown refers, in my view, to humoral responses.

It is possible that an antibody-dependent immune response controls parasite numbers and as such less immune cell effector function is promoted, and not only activation of immunosuppressive signatures

The mouse data show low parasitamieas following an acute infection while the AM human data is likely the product of infection continuously remaining below the clinical threshold. I recommend infection mice with much lower number of iRBCs to better mimic an asymptomatic infection, or to be more cautious regarding its interpretation.

the authors do a good job in referring to work showing expression data of previous studies, and this reviewer agrees that there is

a lack in RNAseq data of AM, but the reference cited Boldt et al PMID: 30638864 includes asymptomatic infection and could be mentioned and maybe mentioning immune phenotypic done through other techniques as done by Andrade et al. PMID: 33106664 could be worth discussing.

Minor commnents
There is a new who malaria report from 2021, shall the authors want to refer top the latest numbers

Portugal et al, 2017 shows only that treating children right before the season starts has no effect on clinical malaria risk, but it remains unclear if immunomodulatory effects would require more time to fade away after clearance, and this could be more clearly transmitted

Please indicate whether mean and SD, median and IQR, or whatever it is shown in figure legends.

Indicate also what the dashed line on Fig 1G shows and how background was controlled for in the legend or methods section.

If there are significant differences in Fig. 3 these should be identified in the Figure.

In the section "Asymptomatic P. falciparum malaria drives a transcriptional profile supporting immunosuppressive processes" it seems that Fig 6C is wrongly called 5C, please correct.

Legend of Figures with Chord diagrams should be more complete and better guide the reader to what connection are highlighted in the text and what are represent different colours on the inner circle.

We sincerely thank the reviewers for taking the time to assess our manuscript. We found the feedback very useful in improving to the delivery of our study. We welcome the suggestion to elaborate more on the genes differentially expressed between asymptomatic individuals and healthy controls, which has now allowed us to bring to the surface important information hidden in the analysis.

Reviewer #1:

Studniberg et al. used a combination of systems biology tools and analysis to characterize differences among healthy individuals, symptomatic malaria, and asymptomatic malaria and found several parameters that correlate with disease severity. In addition, they discovered an immunosuppressive transcriptional signature with upregulation of pathways involved in the inhibition of T cell function, and CTLA-4. They then further validated this finding in mouse model of malaria. This is a well-designed and executed study and the manuscript is well written as well. I only have some minor questions and suggestions.

We thank the reviewer por the positive appraisal of our manuscript.

1. For Figure 1G, they need to include or discuss the Pf antibody titer in normal healthy individuals, not living in those endemic area.

Thanks, for the comment. Antibody levels of malaria-naïve healthy Melbourne controls were shown as a dotted line in Fig 1G. This has now been clarified in the figure legend.

2. Their PD-1 cytof signal is low. Therefore, I think it is necessary to show that the antibody itself is working.

Although the PD-1 CyTOF signal is low compared to other markers in the panel, the metal-labelled antibody used in our stainings produces highly reproducible results. Similar populations of CD27$^+$PD-1$^+$ T$_{H1}$-like memory CD4$^+$ T cells and CXCR3$^+$T-bet$^+$PD-1$^+$ memory T$_{FH}$ cells have been detected in the present manuscript as wells as in *P. vivax*-infected individuals in a recently published study (JCI Insight 2021;6(14):e148086). We have confirmed the expression of PD-1 in these cells by manual gating. An example showing percentage of PD-1$^+$ cells among gated CD3$^+$ cells (top) and PD-1 expression among gated CD4$^+$ T$_{H1}$ cells (bottom) is provided in the reviewer's figure.

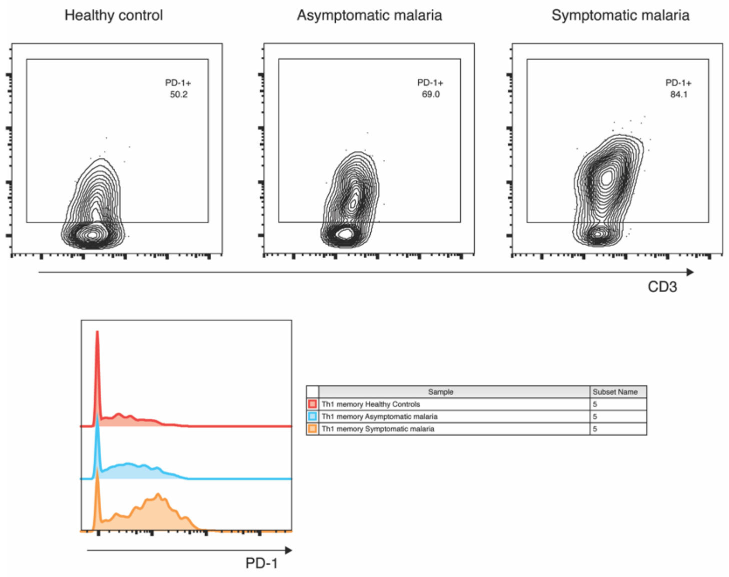

3. Figure 3B missing label for significance.

The results in Figs 3A-E were generated using the CITRUS algorithm in Cytobank. CITRUS is an algorithm designed for the fully automated discovery of statistically significant stratifying biological signatures within single cell datasets containing numerous samples across multiple known endpoints. CITRUS performs unsupervised hierarchical clustering to identify clusters of cellular populations within the overall dataset. CITRUS then uses a regularized supervised learning algorithm to determine the populations and features that best predict differences across groups. A false discovery rate (FDR) is set up before the algorithm starts to run the analysis. Our analysis was performed at an FDR of 5% ($p<0.05$). More information has been provided both in the Results section and figure legend to be better explain how CITRUS discovers significantly different populations across groups.

4. I think it would be interesting to discuss a literature survey on whether people with asymptomatic malaria would have a poor response rate to regular vaccines given that they have a immunosuppressive signature.

Thank you for this excellent suggestion. We have included in the discussion examples of attenuated sporozoite immunisation results, showing that vaccine immunogenicity is significantly reduced in malaria-exposed African adults compare to malaria-naïve controls.

Reviewer #2:

This is an interesting paper that contains a goldmine of data and hints at some novel inflammatory, metabolic and immune pathways associated with malaria infection. However, in its current format it presents a somewhat confusing, and at times contradictory, story. However, with a major rewrite, and perhaps a reframing of the underlying hypotheses, it could make a valuable contribution to the literature.

Thank you for acknowledging the potential of our study. We have taken the reviewer's comments onboard, and we trust that this revised version provides a more balanced delivery of our findings.

Firstly, the title is somewhat misleading and fails to capture the full extent of the observations presented. One aspect of the data (relating to AM) seems to have been plucked out of the many observations and highlighted, over emphasising one aspect of the data at the expense of the rest.

We agree with the reviewer. Our proposed title was longer and had to be shortened to meet editorial requirements. In this revised version, we have changed it to "**Molecular profiling reveals an immunosuppressive signature in asymptomatic *P. falciparum* malaria**", which is under 100 words.
We will be happy to change the tittle to "**Molecular profiling reveals features of clinical immunity and immunosuppression in asymptomatic *P. falciparum* malaria",** that better captures all responses found in the study, provided editorial approval.

The focus, in the introduction and discussion, is on the potential deleterious consequences of asymptomatic malaria infections. The appropriate comparator group for such analysis is with the healthy controls, as any intervention would aim to convert these people to a healthy state, however in many of the analyses the primary comparisons are between AM and SM or between SM and HC, with much less attention paid to the AM/HC comparison. Indeed, the comparison of RNAseq data between AM and HC is only presented in the supplementary figures, not in the main text. I would argue that there are three valid comparisons to be made in this analysis: AM vs HC, AM vs SM and SM vs HC. All three should be shown wherever possible. Comparison of AM with HC is essential to determine whether AM is deleterious.

The reviewer raised an important point. We have now moved the contrast between AM vs HC out of EV and into a new primary figure (Figure 7). We elaborated a lot more on various immunoregulatory pathway impacted by asymptomatic infection. Furthermore, we have included examples of specific immunoregulatory genes which are equally upregulated or downregulated by asymptomatic malaria compared to both symptomatic individuals and healthy controls (Figure 7E).

Throughout the paper, the authors refer to pathways that down regulate T cell effector functions as "immunosuppressive". Another way to think of it is that these responses are "immunoregulatory" and thus supportive of homeostasis, rather than "immunosuppressive" which implies that they are in some way pathological. The authors have not really presented any data to demonstrate that down regulation of the T cell response is detrimental.

Thanks for the comment. To further the analysis around this topic we have now looked at correlations of immunoregulatory genes differentially expressed between asymptomatic vs symptomatic individuals and parasitemia. We have found two groups of genes, some that show significant correlations with parasitemia and some that did not. We hypothesized that changes in transcriptional profiles of immunoregulatory genes as a function of high parasitemia could be driven by high parasite burdens and/or the concomitant inflammation associated with symptomatic infection, and presumably respond to homeostatic requirements. In contrast, we suggest that changes in transcriptional profiles not correlated with high parasitemia, might result from persistent clinicallysilent infections. These concepts are now included in the manuscript.

Old Figure 8 (now Figure 9) provides proof of concept for the T cell suppressive-CTLA-4 pathway in the control of asymptomatic parasitemia. Furthermore, we also show identified similar sources of CTLA-4 expression during asymptomatic infection in rodent and *P. falciparum* human malaria. It is possible that immunosuppressive processes arise as a homeostatic mechanism to prevent inflammation-driven pathology during persistent asymptomatic infection, but at the expense of reducing the capacity of the cellular response to fully control parasitemia. This concept has been added to the discussion.

On a related point, there is some rather simplistic thinking . For example, the implication that upregulation of inflammatory pathways that mediate disease symptoms will automatically lead to inhibition of acquisition of antibody-mediated immunity. This assumes that the concentrations of cytokines mediating inflammation are the same as those that modulate antibody responses. This is not necessarily the case and it is at least plausible that one outcome can occur independently of the other depending on the timing, concentration and wider environment of the response.

Thank you for pointing this out. The concept that IFN-gamma-mediated responses modulate the acquisition of humoral immunity arises from a large body of data in humans and mice demonstrating an important upregulation of the T_{H1}-defining transcription factor T-bet in T_{FH} cells, which results in diminished helper capacity. We fully agree with the reviewer that the role of inflammatory pathway in the control of humoral response is complex than that our data shows that too. For example, we found that T-bet plays a dual role in infection. While T-bet expression in T_{FH} cells impairs their differentiation, thereby reducing the magnitude of the antibody response, T-bet expression in B cells promotes the differentiation of cells with increased affinity for antigen, thereby improving the quality of the antibody response. This has now been incorporated into the Introduction.

This cross-sectional study design does not allow causality to be ascribed to any of the observations and certainly does not allow the direction of any supposed causal relationship to be determined. In order to begin to demonstrate causality, and to predict which parameter predicts which, you need a longitudinal study design and/or corroboration of the observational data with experimental data. The corroboration of the CTLA-4 data in the mouse model is a very nice example of this, but it is the only example.

The manuscript is littered with comments suggesting causality and the direction of causality, e.g :
Page 8, line 7: "PD-1 +CXCR3+ T-bet+ TFH cells predicted increased susceptibility to P. falciparum symptomatic malaria
P8 line 9: "classical and activated MBCs were associated with increased risk of

symptomatic infection
Page 8 Line 13 "associated with protection from symptomatic infection .....Thus, diverse MBCs expressing low chemokine receptors levels and a CD4+ TH2 cell bias predict reduced risk of clinical malaria.
Page 12. Line 8 "downregulated by asymptomatic malaria"

We acknowledge the limitations of a not longitudinal follow up. We have extensively revised the manuscript and endeavoured to find a more balance wording to describe the results. In some experiments, we performed logistic regression and obtained statistically significant odds ratios. By definition, odds ratios give information on the probability or risk of an event occurring or not, even in not longitudinal settings. We have rephrased as required to make sure that our wording reflects that.

P12, Line 21 "indicate that low parasitemia asymptomatic P. falciparum infections activate a blood transcriptional profile that drives several immunosuppressive processes" (This could be rephrased as a hypothesis rather than stated as a fact).

This sentence has been largely rephrased in the context of our new correlation analysis of transcriptional profiles with parasitemia levels. In brief, we now say …" raising the possibility that low parasitemia asymptomatic *P. falciparum* infections activate a blood transcriptional profile that drives immunosuppressive processes"…

Page 19 line 12 and 14; upregulated by asymptomatic infection"; "asymptomatic malaria induced the upregulation of NR1D1 "
There are many more. The entire ms. needs to be carefully checked and these statements of association or prediction removed or qualified.

The manuscript was thoroughly checked and rephrased to achieve a more balanced delivery of our findings.

I have some concerns over the study design that limit the utility of the data. No consideration is given to these limitations in either the presentation or interpretation of the data. For example,
• this is an opportunistic study based on stored samples that were not collected with this purpose in mind. To what extent has this hampered the execution of the study? Were there limitations in cell numbers, cell quality (the cells have been stored for more than a decade, how were they quality controlled?), RNA quality etc that have limited the number of assays performed.

The study was specifically set up in the Timika region for the purpose of the current study: identify parameters associated with clinical immunity to malaria We have recently published the *P. vivax* arm of this study in a recent paper (Ioannidis et al, JCI insight, 2021). Samples were collected by Indonesian staff in Timika, who prepared plasma and PBMC that were rapidly frozen and stored in liquid nitrogen. Cell and RNA quality was good. Strict ethics protocol in Indonesia allowed collection of no more than ~ 5-10 ml of blood from consenting participants. On occasions, no enough material was obtained to run all endpoints proposed in the study, which explains the smaller subset employed for RNA-sequencing.

• only a subset of samples were analysed by RNA seq. How were these samples selected? On the basis of sample quality? If so, how comparable are the groups included in the RNA

seq analysis (data for the full cohort is shown in Fig 1; we need to see the same data just for the subset included in the RNA seq analysis).

Samples were selected for RNA-seq on the basis on material abundance. Samples with larger cell number, with enough material to run CyTOF and RNA-seq were used for this analysis. Clinical parameters (parasitemia, hematocrit, etc), a range of antibody responses and cell populations identified by CyTOF of the subgroup used for RNA-seq were representative of values observed across the larger cohort. We have created a new figure (Figure EV2) showing that there were no significant differences across these endpoints between the average of the full cohort and the sub-group used for transcriptomics analysis.

• *whilst the groups are roughly comparable, overall, the age range is very broad. Have you checked to see whether there are any age effects on any of the analyses?*

Age was not found to be a confounder in this cohort. Papuans reside both in the Timika lowlands where malaria exposure is common and the highlands where malaria is absent. Migration of non-immune adults from the highlands to lowlands means a first malaria infection (often symptomatic) can occur in all age groups. This is a highly unique scenario, compared to many settings in Africa, in which younger people develop symptomatic infection and adults are asymptomatic carriers, and age is clearly a confounder. This information has been added to the study design.

• *similarly, the range of parasitemias is quite high (and overlapping) between AM and SM. Have the data been checked for any association with parasitemia?*

Parasitemia was not a confounder in the cohort. By study design, symptomatic individuals are by definition higher in parasitemia than asymptomatic ones. As stated in the methods all individuals included in the analysis of immune response had >500 parasite/mL blood.

As discussed above, we have looked at specific correlations of selected immunoregulatory genes differentially expressed between AM vs SM and parasitemia and found that whereas most transcriptional profiles are not correlated with parasitemia, a few genes showed negatively associated with high parasitemia. The implication of the findings is discussed in manuscript, as mentioned above.

Fig 3 (and elsewhere) - why are all 3 groups not shown side by side on the same bar charts? Difference between AM and HC is as important as differences between AM vs SM.

The results in Figs 3A-E were generated using the CITRUS algorithm in Cytobank. CITRUS is an algorithm designed for the fully automated discovery of statistically significant stratifying biological signatures within single cell datasets containing numerous samples across multiple known endpoints. CITRUS performs unsupervised hierarchical clustering to identify clusters of cellular populations within the overall dataset. CITRUS then uses a regularized supervised learning algorithm to determine the populations and features that best predict differences across groups. That is why some populations were identified as differentially abundant between HC and SM and not necessarily between AM and SM and vice verse.
A false discovery rate (FDR) is set up before the algorithm starts to run the analysis. Our analysis was performed at 5%FDR ($p<0.05$). More information has been provided both in the

Results section and figure legend to be better explain how CITRUS discovers significantly different populations across groups.
Throughout the manuscript, differences are reported as being statistically significant but the statistical data are not provided. Stats should be given (in the text or figures) for all differences that are claimed to be significant, and should be adjusted for age and parasitemia if necessary.

No confounders were identified in the cohort. We have corrected all stats missing throughout the figure legends.

Finally, the authors present some interesting and plausible hypotheses relating to upregulation and down regulation of different pathways. It would be really helpful - and would greatly increase the accessibility of the findings - if these ideas could be summarised in a graphical summary or graphical abstract.

Excellent suggestion. The revised version includes a synopsis image.

Reviewer #3:

Studniberg and colleagues investigate immune phenotypes of healthy uninfected individuals, and P. falciparum infected individuals with and without malaria symptoms by mass cytometry and RNAseq and antibody responses, and search for associations. While the work is well done a presented I provide a few ideas, that I believe will help improve the manuscript.

We thank the reviewer for the positive appraisal of our manuscript.

The authors often refer to cell populations predicting increased or decreased susceptibility to P. falciparum symptomatic malaria, but their data is associative only. Individuals showing symptoms or not at the time of cell collection only, without any longitudinal data. Although interesting, i believe the wording should be revised. For instance MBCs expressing low chemokine receptors were more commonly seed in individuals without clinical malaria, but they might not be predictive of protection, and only be more expressed once symptoms arise.

Thank you for the fair suggestion. We acknowledge the limitations of a not longitudinal follow up. We have extensively revised the manuscript and endeavoured to find a more balance wording to describe the results. In some experiments, we performed logistic regression and obtained statistically significant odds ratios. By definition, odds ratios give information on the probability or risk of an event occurring or not, even in not longitudinal settings. We have rephrased as required to make sure that our wording reflects that.

I believe comparisons shown in Fig 3 would gain from showing all 3 population and not only two, it is not clear why some show HC and SM, while other show AM and SM.

The results in Figs 3A-E were generated using the CITRUS algorithm in Cytobank. CITRUS is an algorithm designed for the fully automated discovery of statistically significant stratifying biological signatures within single cell datasets containing numerous samples across multiple known endpoints. CITRUS performs unsupervised hierarchical clustering to identify clusters of cellular populations within the overall dataset. CITRUS then uses a regularized supervised learning algorithm to determine the populations and features that best predict differences across groups. That is why some populations were identified as differentially abundant between HC and SM and not necessarily between AM and SM.
A false discovery rate (FDR) is set up before the algorithm starts to run the analysis. Our analysis was performed at a 5%FDR ($p<0.05$). More information has been provided both in the Results section and figure legend to be better explain how CITRUS discovers significantly different populations across groups.

How was the number samples used for PBMC RNAseq decided and how were these selected from the larger pool? this should be clear in the methods or result section.

Samples were selected for RNA-seq on the basis on material abundance. Samples with larger cell number, with enough material to run CyTOF and RNA-seq (as well as other endpoints not shown in the manuscript) were used for this analysis. Our preliminary experiments indicated that ~6 samples/group were enough to provide good segregation of transcriptional profiles at a 15%FDR. This information has been included in the text.

How did the 5 or 6 samples included compare to the rest in terms of parasitemia and T and B cell subpopulations. Fig 3 could easily show the proportions of the different CD4 and MBC subpopulations and then identify the 5 or 6 samples use din Fig 4.

Clinical parameters (parasitemia, hematocrit, etc), a range of antibody responses and cell populations identified by CyTOF of the subgroup used for RNA-seq were representative of values observed across the larger cohort. We have created a new figure (Figure EV2) showing that there were no significant differences across these endpoints between the average of the full cohort and the sub-group used for transcriptomics analysis.

this would also allow better discussing if RNAseq differences observed in Fig 4are likely to be promoted by different proportions of cell types, and support or not the statement "suggesting that transcriptional differences detected downstream would reflect changes in cell activity between clinical groups rather than fluctuations in the composition of the PBMC pool." which reads too strong to me without more data rot back it.
Furthermore, up-regulation of terms involved in cell proliferation in symptomatic malaria by the RNAseq data might point to increase of particular subpopulations

Thanks for pointing this out. That is correct. New Figure 8 (old Fig 7) shows a correlation between terms involved in proliferation and IgM^+ activated MBC, abundant among symptomatic individuals. The d-tangle package only infers broad populations (like total MBC, $CD4^+$, $CD8^+$ T cells) and fluctuations in smaller subsets cannot be detected. The statement has been deleted.

Why are the FDRs in Fig 4 C and D different? How many DEG would be founf if FDR would would 5% and not 15 in Fig 4C.

That was a mistake and we thank the reviewer for pointing this out. We have corrected Fig 4D using a 15%FDR, consistent with Fig 4C. It is well documented (Idaghdour *et al*, 2012; Lee *et al*, 2018; Tran *et al*, 2016)that detection of blood transcriptional activity in response to infection, diminishes with lower parasitemia levels. Thus, a 15%FDR was used to improve detection of transcribed genes during low parasitemia asymptomatic infection.

The authors could try to identity the cell populations/pathways promoting the gene expression differences between AM and SM, but should be cautious and have into account if gene expression differences between AM and HC are present (as in cluster 4 of Fig 6c, otherwise differences between AM and SM might be just a product of differences between symptoms or not. Accordingly, in my view some of Fig EV2 should be made available in the main manuscript figures.
In my view the authors should be caustics as to define direction and causation of the association seen in these data.

The reviewer raised an important point. We have now moved the contrast between AM vs HC out of EV and into a new primary figure (Figure 7). We elaborated a lot more on various immunoregulatory pathway impacted by asymptomatic infection. Furthermore, we have included examples of specific immunoregulatory genes which are equally upregulated or downregulated by asymptomatic malaria compared to both symptomatic individuals and healthy controls (Figure 7E).

In Figure 6, we have now looked at correlations of immunoregulatory genes differentially expressed between asymptomatic vs symptomatic individuals and parasitemia. We have found two groups of genes, some that show significant correlations with parasitemia and some that did not. We hypothesized that changes in transcriptional profiles of immunoregulatory genes as a function of parasitemia could be driven by high parasite burdens and/or the concomitant inflammation associated with symptomatic infection. In contrast, we propose that changes in transcriptional profiles not correlated with high parasitemia, are more likely to be the result of persistent clinically-silent infections. These concepts are now included in the manuscript.

I recommend altering the title of section "Asymptomatic P. falciparum malaria supports humoral responses to infection while driving cell mediated immunosuppressive responses" and very little if anything on the data shown refers, in my view, to humoral responses.

The title was changed to “Asymptomatic *P. falciparum* malaria-immunosuppressive transcriptional profiles are not correlated responses associated with reduced risk of symptomatic infection”

It is possible that an antibody-dependent immune response controls parasite numbers and as such less immune cell effector function is promoted, and not only activation of immunosuppressive signatures

Yes. We agree. It is possible that there is no need to mount a high cellular response when there are high antibody levels. However, asymptomatic infections are persistent and difficult to self-resolve, even with good antibody levels, suggesting an additional role for T cell responses in immunity. Our analysis uncovered several anti-proliferative and anti-inflammatory pathways upregulated during these infections and using a mouse infection model we provided proof of concept that blockade of one of these pathways (CTLA-4) facilitates the control of asymptomatic parasitemia. It is therefore possible that immunosuppressive mechanisms arise to prevent inflammation-driven pathology during persistent infections but at the expense of reducing the capacity of the cellular response to fully control parasitemia. We have added this concept to the discussion.

The mouse data show low parasitamieas following an acute infection while the AM human data is likely the product of infection continuously remaining below the clinical threshold. I recommend infection mice with much lower number of iRBCs to better mimic an asymptomatic infection, or to be more cautious regarding its interpretation.

The infection model does not shield reproducible results using infections with much lower number of iRBCs. We have rephrased the text to acknowledge the limitations of the mouse infection model and the differences with human malaria.

the authors do a good job in referring to work showing expression data of previous studies, and this reviewer agrees that there is a lack in RNAseq data of AM, but the reference cited Boldt et al PMID: 30638864 includes asymptomatic infection and could be mentioned and maybe mentioning immune phenotypic done through other techniques as done by Andrade et al. PMID: 33106664 could be worth discussing.

Thanks for the suggestion. The two references have been added to the discussion.

Minor commnents

There is a new who malaria report from 2021, shall the authors want to refer top the latest numbers

The 2021 WHO malaria report in now quoted.

Portugal et al, 2017 shows only that treating children right before the season starts has no effect on clinical malaria risk, but it remains unclear if immunomodulatory effects would require more time to fade away after clearance, and this could be more clearly transmitted

The text in the introduction and discussion has been modified.

Please indicate whether mean and SD, median and IQR, or whatever it is shown in figure legends.

We have including all missing statistical parameters from the figure legends. Thank you.

Indicate also what the dashed line on Fig 1G shows and how background was controlled for in the legend or methods section.

Antibody levels of malaria-naïve healthy Melbourne controls were shown as a dotted line in Fig 1G. This has now been clarified in the figure legend.

If there are significant differences in Fig. 3 these should be identified in the Figure.

As discussed above, CITRUS is an algorithm designed for the fully automated discovery of statistically significant signatures within single cell datasets. A false discovery rate is set up before the algorithm starts to run the analysis. Our analysis was performed at a 5%FDR (p>0.05). This is the p value for all identified populations. This has been clarified in the text.

In the section "Asymptomatic P. falciparum malaria drives a transcriptional profile supporting immunosuppressive processes" it seems that Fig 6C is wrongly called 5C, please correct.

Thank you. This has been corrected.

Legend of Figures with Chord diagrams should be more complete and better guide the reader to what connection are highlighted in the text and what are represent different colours on the inner circle.

We have included extra labelling in the chord diagrams for clarity. As we now elaborated more on the genes differentially expressed between asymptomatic infection and controls, relevant genes from that group have been incorporated into the chord too (New Figure 8D).

Point-by-Point to the editors queries MSB-2021-10824R Initial Quality Check

We would like to thank the editors very much for the outstanding quality check of our manuscript. Please find our point-by-point response to the editor queries below:

** Pre-acceptance checks by our data editors have raised a few queries in the manuscript which you will find as comments in the attached edited/commented Word document with "Track changes" activated. We would appreciate if you incorporated the requested final text modifications directly into this attached version, uploading the edited document upon re-submission with changes/additions still highlighted via the "Track changes" option. This will help us to facilitate our final checking.*

We have addressed all comments within the attached manuscript and have highlighted all changes via the track changes option. The finalised manuscript has been re-uploaded to the portal.

** Dataset GSE181179 is not accessible/available, please provide access for referees/editor. Please also remember that all datasets must be publicly accessible latest upon acceptance.*

Dataset GSE181179 has now been made publicly accessible.

** Figure 4. The legend describes A-D but the figure displays A-E. Figure 4E is also not listed in the figure legend or called out in the manuscript.*

This was our mistake as we had uploaded an old version of figure 4. The new version that does not include a figure 4E has been re-uploaded.

** During a standard image analysis we detected potential duplications in the figure set, 2 A-E and 3 A-E. We would like you to clarify this issue before sending your paper back to referees. Please update the respective figure legends or if you make changes to the figures, please include a point-by-point describing what you have changed.*

We have repeated t-SNE panels in figures 2A-E and in figures 3A-E intentionally to facilitate interpretation of figure 3. However, we will be happy to remove the repeated t-SNE panels in figures 3A-E if the editors consider them to be redundant.

22nd Mar 2022

RE: MSB-2021-10824R, Molecular profiling reveals features of clinical immunity and immunosuppression in asymptomatic P. falciparum malaria

Dear Prof. Hansen,

Thank you for sending us your revised manuscript. We have now heard back from reviewer #3 who was asked to evaluate your revised study. As you will see below, they think that the reviewers' concerns have been satisfactorily addressed. I am glad to inform you that we can soon accept the study for publication, pending some minor editorial issues listed below, as well as a minor issue listed by the reviewer.

- Please make all requested text changes using the attached .doc file and *keeping the "track changes" mode* so that we can easily access the edits made.

- Benediktus Andries, Pak Prayoga, Novita Sariyanti, Wei Shi, Alexandra L. Garhnham are missing from the "Author Contributions". Please add this information.

- There is a callout to Supplementary Table 1 but no table is provided. Please check. If a table is provided it should be labeled and called out as table Ev1.

- Our data integrity analyst noted that Figure panels 2A-E are reused in Figure 3A-E. We would ask you to clearly indicate the data/panel reuse in the respective figure legends for transparency.

- We agree with using the title "Molecular profiling reveals features of clinical immunity and immunosuppression in asymptomatic P. falciparum malaria", you can update the text accordingly.

- The provided synopsis image is rather detailed and does not display well at the required final size (i.e. 500 px width). Please provide an updated image, exactly at this size (width = 500 px, height max. 500 px), and make sure that all labels are readable. All text (besides labels) should be removed from the synopsis image.

Please resubmit your revised manuscript online **within one month** and ideally as soon as possible. If we do not receive the revised manuscript within this time period, the file might be closed and any subsequent resubmission would be treated as a new manuscript. Please use the Manuscript Number (above) in all correspondence.

Click on the link below to submit your revised paper.

Link Not Available

Thank you for submitting this paper to Molecular Systems Biology.

Yours sincerely,

Maria Polychronidou, PhD
Senior Editor
Molecular Systems Biology

Reviewer #3:

Studniberg and colleagues' reply to my and other reviewers comments has improved the manuscript, and I have no further questions.

Very minor suggestion, I would include the number of subjects included in fig 1 and referred on the on the figure legend, also in the result description.

The authors performed the requested editorial changes.

31st Mar 2022

RE: MSB-2021-10824RR, Molecular profiling reveals features of clinical immunity and immunosuppression in asymptomatic P. falciparum malaria

Dear Diana,

Thank you again for sending us your revised manuscript. We are now satisfied with the modifications made and I am pleased to inform you that your paper has been accepted for publication.

*** PLEASE NOTE *** As part of the EMBO Publications transparent editorial process initiative (see our Editorial at https://dx.doi.org/10.103/msb.2010.72), Molecular Systems Biology publishes online a Review Process File with each accepted manuscripts. This file will be published in conjunction with your paper and will include the anonymous referee reports, your point- by-point response and all pertinent correspondence relating to the manuscript. If you do NOT want this File to be published, please inform the editorial office at msb@embo.org within 14 days upon receipt of the present letter.

LICENSE AND PAYMENT:
All articles published in Molecular Systems Biology are fully open access: immediately and freely available to read, download and share.

Molecular Systems Biology charges an article processing charge (APC) to cover the publication costs. You, as the corresponding author for this manuscript, should have already received a quote with the article processing fee separately. Please let us know in case this quote has not been received.

Once your article is at Wiley for editorial production, you will receive an email from Wiley's Author Services system, which will ask you to log in and will present you with the publication license form for completion. Within the same system the publication fee can be paid by credit card, an invoice or pro forma can be requested.

Payment of the publication charge and the signed Open Access Agreement form must be received before the article can be published online.

Upon acceptance it is mandatory for you to return the completed payment form. Failure to send back the form may result in a delay in the publication of your paper.

Molecular Systems Biology articles are published under the Creative Commons licence CC BY, which facilitates the sharing of scientific information by reducing legal barriers, while mandating attribution of the source in accordance to standard scholarly practice.

Proofs will be forwarded to you within the next 2-3 weeks.

Thank you very much for submitting your work to Molecular Systems Biology.

Kind regards,

Maria

Maria Polychronidou, PhD
Senior Editor
Molecular Systems Biology

EMBO Press Author Checklist

Corresponding Author Name: Diana S. Hansen	
Journal Submitted to: Molecular Systems Biology	
Manuscript Number: MSB-2021-10824	

USEFUL LINKS FOR COMPLETING THIS FORM
The EMBO Journal - Author Guidelines
EMBO Reports - Author Guidelines
Molecular Systems Biology - Author Guidelines
EMBO Molecular Medicine - Author Guidelines

Reporting Checklist for Life Science Articles (updated January 2022)
This checklist is adapted from Materials Design Analysis Reporting (MDAR) Checklist for Authors. MDAR establishes a minimum set of requirements in transparent reporting in the life sciences (see Statement of Task: 10.31222/osf.io/9sm4x). Please follow the journal's guidelines in preparing your manuscript.
Please note that a copy of this checklist will be published alongside your article.

Abridged guidelines for figures
1. Data
The data shown in figures should satisfy the following conditions:
- the data were obtained and processed according to the field's best practice and are presented to reflect the results of the experiments in an accurate and unbiased manner.
- ideally, figure panels should include only measurements that are directly comparable to each other and obtained with the same assay.
- plots include clearly labeled error bars for independent experiments and sample sizes. Unless justified, error bars should not be shown for technical replicates.
- if n<5, the individual data points from each experiment should be plotted. Any statistical test employed should be justified.
- Source Data should be included to report the data underlying figures according to the guidelines set out in the authorship guidelines on Data Presentation.

2. Captions
Each figure caption should contain the following information, for each panel where they are relevant:
- a specification of the experimental system investigated (eg cell line, species name).
- the assay(s) and method(s) used to carry out the reported observations and measurements.
- an explicit mention of the biological and chemical entity(ies) that are being measured.
- an explicit mention of the biological and chemical entity(ies) that are altered/varied/perturbed in a controlled manner.
- the exact sample size (n) for each experimental group/condition, given as a number, not a range;
- a description of the sample collection allowing the reader to understand whether the samples represent technical or biological replicates (including how many animals, litters, cultures, etc.).
- a statement of how many times the experiment shown was independently replicated in the laboratory.
- definitions of statistical methods and measures:
  - common tests, such as t-test (please specify whether paired vs. unpaired), simple χ2 tests, Wilcoxon and Mann-Whitney tests, can be unambiguously identified by name only, but more complex techniques should be described in the methods section;
  - are tests one-sided or two-sided?
  - are there adjustments for multiple comparisons?
  - exact statistical test results, e.g., P values = x but not P values < x;
  - definition of 'center values' as median or average;
  - definition of error bars as s.d. or s.e.m.

Please complete ALL of the questions below.
Select "Not Applicable" only when the requested information is not relevant for your study.

Materials

Newly Created Materials	Information included in the manuscript?	In which section is the information available? (Reagents and Tools Table, Materials and Methods, Figures, Data Availability Section)
New materials and reagents need to be available; do any restrictions apply?	Not Applicable	

Antibodies	Information included in the manuscript?	In which section is the information available? (Reagents and Tools Table, Materials and Methods, Figures, Data Availability Section)
For **antibodies** provide the following information:<br>- Commercial antibodies: RRID (if possible) or supplier name, catalogue number and or/clone number<br>- Non-commercial: RRID or citation	Yes	Reagents ant Tools Table

DNA and RNA sequences	Information included in the manuscript?	In which section is the information available? (Reagents and Tools Table, Materials and Methods, Figures, Data Availability Section)
**Short novel DNA or RNA including primers, probes**: provide the sequences.	Not Applicable	

Cell materials	Information included in the manuscript?	In which section is the information available? (Reagents and Tools Table, Materials and Methods, Figures, Data Availability Section)
**Cell lines:** Provide species information, strain. Provide accession number in repository **OR** supplier name, catalog number, clone number, and/**OR** RRID.	Not Applicable	
**Primary cultures:** Provide species, strain, sex of origin, genetic modification status.	Not Applicable	
Report if the cell lines were recently **authenticated** (e.g., by STR profiling) and tested for mycoplasma contamination.	Not Applicable	

Experimental animals	Information included in the manuscript?	In which section is the information available? (Reagents and Tools Table, Materials and Methods, Figures, Data Availability Section)
**Laboratory animals or Model organisms:** Provide species, strain, sex, age, genetic modification status. Provide accession number in repository **OR** supplier name, catalog number, clone number, **OR** RRID.	Yes	Reagents and Tools Table
**Animal observed in or captured from the field:** Provide species, sex, and age where possible.	Not Applicable	
Please detail **housing and husbandry conditions**.	Yes	Material and Methods

Plants and microbes	Information included in the manuscript?	In which section is the information available? (Reagents and Tools Table, Materials and Methods, Figures, Data Availability Section)
**Plants:** provide species and strain, ecotype and cultivar where relevant, unique accession number if available, and source (including location for collected wild specimens).	Not Applicable	
**Microbes:** provide species and strain, unique accession number if available, and source.	Not Applicable	

Human research participants	Information included in the manuscript?	In which section is the information available? (Reagents and Tools Table, Materials and Methods, Figures, Data Availability Section)
If collected and within the bounds of privacy constraints report on age, sex and gender or ethnicity for all study participants.	Yes	Material and Methods, Results and Figures

Core facilities	Information included in the manuscript?	In which section is the information available? (Reagents and Tools Table, Materials and Methods, Figures, Data Availability Section)
If your work benefited from core facilities, was their service mentioned in the acknowledgments section?	Yes	Acknowledgements

Design

Study protocol	Information included in the manuscript?	In which section is the information available? (Reagents and Tools Table, Materials and Methods, Figures, Data Availability Section)
If study protocol has been **pre-registered, provide DOI in the manuscript**. For clinical trials, provide the trial registration number **OR** cite DOI.	Not Applicable	
Report the **clinical trial registration number** (at ClinicalTrials.gov or equivalent), where applicable.	Not Applicable	

Laboratory protocol	Information included in the manuscript?	In which section is the information available? (Reagents and Tools Table, Materials and Methods, Figures, Data Availability Section)
Provide DOI OR other citation details if **external detailed step-by-step protocols** are available.	Not Applicable	

Experimental study design and statistics	Information included in the manuscript?	In which section is the information available? (Reagents and Tools Table, Materials and Methods, Figures, Data Availability Section)
Include a statement about **sample size** estimate even if no statistical methods were used.	Yes	Material and Methods, Results
Were any steps taken to minimize the effects of subjective bias when allocating animals/samples to treatment (e.g. **randomization procedure**)? If yes, have they been described?	Not Applicable	
Include a statement about **blinding** even if no blinding was done.	Not Applicable	
Describe **inclusion/exclusion criteria** if samples or animals were excluded from the analysis. Were the criteria pre-established?<br><br>If sample or data points were omitted from analysis, report if this was due to attrition or intentional exclusion and provide justification.	Not Applicable	
For every figure, are **statistical tests** justified as appropriate? Do the data meet the assumptions of the tests (e.g., normal distribution)? Describe any methods used to assess it. Is there an estimate of variation within each group of data? Is the variance similar between the groups that are being statistically compared?	Yes	Material and Methods, Results, Figure Legends

Sample definition and in-laboratory replication	Information included in the manuscript?	In which section is the information available? (Reagents and Tools Table, Materials and Methods, Figures, Data Availability Section)
In the figure legends: state number of times the experiment was **replicated** in laboratory.	Not Applicable	
In the figure legends: define whether data describe **technical or biological replicates**.	Yes	Figure legends where applicable

Ethics

Ethics	Information included in the manuscript?	In which section is the information available? (Reagents and Tools Table, Materials and Methods, Figures, Data Availability Section)
Studies involving **human participants**: State details of **authority granting ethics approval** (IRB or equivalent committee(s), provide reference number for approval.	Yes	Material and Methods
Studies involving **human participants**: Include a statement confirming that **informed consent** was obtained from all subjects and that the experiments conformed to the principles set out in the WMA Declaration of Helsinki and the Department of Health and Human Services Belmont Report.	Yes	Material and Methods
Studies involving **human participants:** For publication of **patient photos**, include a statement confirming that consent to publish was obtained.	Not Applicable	
Studies involving experimental **animals**: State details of **authority granting ethics approval** (IRB or equivalent committee(s), provide reference number for approval. Include a statement of compliance with ethical regulations.	Yes	Material and Methods
Studies involving **specimen and field samples:** State if relevant **permits** obtained, provide details of authority approving study; if none were required, explain why.	Yes	Material and Methods

Dual Use Research of Concern (DURC)	Information included in the manuscript?	In which section is the information available? (Reagents and Tools Table, Materials and Methods, Figures, Data Availability Section)
Could your study fall under dual use research restrictions? Please check biosecurity documents and list of **select agents and toxins** (CDC): https://www.selectagents.gov/sat/list.htm	Not Applicable	
If you used a select agent, is the security level of the lab appropriate and reported in the manuscript?	Not Applicable	
If a study is subject to dual use research of concern regulations, is the name of the **authority granting approval and reference number** for the regulatory approval provided in the manuscript?	Not Applicable	

Reporting

The MDAR framework recommends adoption of discipline-specific guidelines, established and endorsed through community initiatives. Journals have their own policy about requiring specific guidelines and recommendations to complement MDAR.

Adherence to community standards	Information included in the manuscript?	In which section is the information available? (Reagents and Tools Table, Materials and Methods, Figures, Data Availability Section)
State if relevant guidelines or checklists (e.g., **ICMJE, MIBBI, ARRIVE, PRISMA**) have been followed or provided.	Not Applicable	
For **tumor marker prognostic studies,** we recommend that you follow the **REMARK** reporting guidelines (see link list at top right). See author guidelines, under 'Reporting Guidelines'. Please confirm you have followed these guidelines.	Not Applicable	
For **phase II and III randomized controlled trials**, please refer to the **CONSORT** flow diagram (see link list at top right) and submit the CONSORT checklist (see link list at top right) with your submission. See author guidelines, under 'Reporting Guidelines'. Please confirm you have submitted this list.	Not Applicable	

Data Availability

Data availability	Information included in the manuscript?	In which section is the information available? (Reagents and Tools Table, Materials and Methods, Figures, Data Availability Section)
Have **primary datasets** been deposited according to the journal's guidelines (see 'Data Deposition' section) and the respective accession numbers provided in the Data Availability Section?	Yes	Data availability section
Were **human clinical and genomic datasets** deposited in a public access-controlled repository in accordance to ethical obligations to the patients and to the applicable consent agreement?	Not Applicable	
Are **computational models** that are central and integral to a study available without restrictions in a machine-readable form? Were the relevant accession numbers or links provided?	Not Applicable	
If publicly available data were reused, provide the respective **data citations in the reference list**.	Not Applicable	
